# Site of the Hydroxyl Group Determines the Surface Behavior of Bipolar Chain-Oxidized Cholesterol Derivatives—Langmuir Monolayer Studies Supplemented with Theoretical Calculations

**DOI:** 10.1021/acs.jpcb.2c08629

**Published:** 2023-02-23

**Authors:** Anna Chachaj-Brekiesz, Anita Wnętrzak, Jan Kobierski, Aneta D. Petelska, Patrycja Dynarowicz-Latka

**Affiliations:** † Faculty of Chemistry, Jagiellonian University, Gronostajowa 2, 30-387 Kraków, Poland; ‡ Department of Pharmaceutical Biophysics, Faculty of Pharmacy, Jagiellonian University Medical College, Medyczna 9, 30-688 Kraków, Poland; § Faculty of Chemistry, University of Białystok, Ciołkowskiego 1K, 15-425 Bialystok, Poland

## Abstract

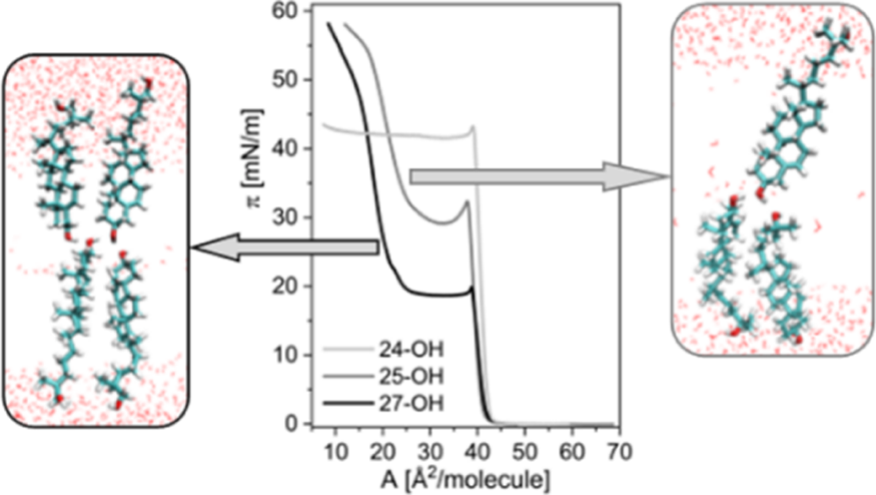

Cholesterol oxidation products (called oxysterols) are involved in many biological processes, showing both negative (e.g., neurodegenerative) and positive (e.g., antiviral and antimicrobial) effects. The physiological activity of oxysterols is undoubtedly closely related to their structure (i.e., the type and location of the additional polar group in the cholesterol skeleton). In this paper, we focus on determining how a seemingly minor structural change (introduction of a hydroxyl moiety at C(24), C(25), or C(27) in the isooctyl chain of cholesterol) affects the organization of the resulting molecules at the phase boundary. In our research, we supplemented the classic Langmuir monolayer technique, based on the surface pressure and electric surface potential isotherms, with microscopic (BAM) and spectroscopic (PM-IRRAS) techniques, as well as theoretical calculations (DFT and MD). This allowed us to show that 24-OH behaves more like cholesterol and forms stable, rigid monolayers. On the other hand, 27-OH, similar to 25-OH, undergoes the phase transition from monolayer to bilayer structures. Theoretical calculations enabled us to conclude that the formation of bilayers from 27-OH or 25-OH is possible due to the hydrogen bonding between adjacent oxysterol molecules. This observation may help to understand the factors responsible for the unique biological activity (including antiviral and antimicrobial) of 27-OH and 25-OH compared to other oxysterols.

## 1 Introduction

Langmuir monolayers of simple amphipathic molecules containing one polar group attached to a hydrophobic moiety have been extensively studied over the years, and the surface properties of such compounds have been well established. Upon the introduction of another polar group, a bipolar molecule is generated. The surface behavior of such molecules is definitely more complex compared to that of the corresponding monopolar amphiphiles, and it was found to depend on the kind and size of polar groups as well as their mutual position in the hydrophobic core. Special attention should be paid to the orientation of bipolar amphiphiles at the free water surface. Overall, there are two different cases to consider: when the two polar groups are either close together or distant from each other.^1,2^ In the first case, the surface behavior of such bipolar molecules is similar to that of single-headed amphiphiles since their polar groups situated nearby act as a single entity. In the latter case, for example, for α,ω-substituted bipolar molecules (the so-called bolaamphiphiles), both polar groups act independently. Within this kind of molecules, different molecular arrangements may appear on the surface of free water depending on the stage of compression. Namely, at low surface pressures/large molecular areas, the molecular organization is similar, that is, molecules lie flat on the surface, fully stretched, with both polar groups submerged in water. However, the differences arise with compression, and the following cases have been described^3^: (i) the molecules do not change their orientation (this may happen for molecules with a rigid hydrophobic core connecting both, usually identical, polar groups) or (ii) the U-shaped (“horseshoe” or “arch”) structure is formed (this happens for molecules having a flexible hydrophobic core). Upon further compression, the molecule may (i) remain with an unchanged orientation until its collapse^4,5^ or (ii) one of the polar groups detaches from the surface, resulting in vertical orientation of a bipolar molecule (one polar group is anchored to the water surface, while the other protrudes toward the air).^6,7^ The latter behavior usually occurs for molecules having distinct polar groups (e.g., hydroxycarboxylic acids^8−11^), although it was also reported in the case of dicarboxylic acids.^12^

It is worth pointing out here that the orientation of molecules at the phase boundary is of utmost importance in their biological activity, and this is closely related to their practical applications. Namely, by compressing a stable Langmuir monolayer to a particular surface pressure, the film can be transferred from the free water surface onto a solid support by the vertical (LB) or horizontal (LS) method. Depending on the target surface pressure, the molecules adopt a particular orientation that is required for application purposes. For example, the deposition of molecules that have one polar group attached to the substrate and the other detached from the surface enables further functionalization of the latter. In addition, in the case of some bioactive molecules, orientation at the phase boundary determines their functioning. For example, gramicidin A is ion conductive but only at surface pressures above 20 mN/m, which corresponds to the vertical orientation of this peptide.^13^ Therefore, the mentioned conditions have to be met for the construction of a biosensor involving this molecule.^14^ The orientation of sterol molecules plays an important role in the formation of lipid rafts. As indicated in theoretical studies,^15^ even a tiny modification in the tetracyclic ring system or in the alkyl chain attached to C(17) may alter the tilt of the molecule, which is related to the effectiveness in increasing the order and condensation of the membrane.

In the literature on Langmuir monolayers from bipolar molecules, most papers report the behavior of those with an aliphatic core while the polycyclic molecules have been studied less frequently. Our attention was drawn to oxysterols, which are biologically active cholesterol derivatives that have an additional oxidized moiety placed either in the fused ring system or in the isooctyl chain.^16,17^ The dual activity of these molecules is intriguing. Namely, in healthy organisms, they are present in very small amounts and play a positive role (primarily in cholesterol homeostasis). Meanwhile, an overproduction of oxidized sterols is associated with many diseases^18^ and pathologies.^19^ The multidirectional activity of oxysterols can lead to opposite effects; for example, they can act carcinogenic and antitumor.^20,21^ It has also been shown that some sterols oxidized in the isooctyl chain (25-hydroxycholesterol (25-OH) and 27-hydroxycholesterol (27-OH)) can act against a wide spectrum of enveloped and nonenveloped viruses.^22,23^ On the contrary, other oxysterols (independent of the additional polar group position in the sterol structure) were found to be significantly less effective in this respect or lacked antiviral activity.

This inspired us to undertake studies on the physiologically crucial oxysterols having an additional hydroxyl group in the octyl chain, that is, at C(24), C(25), and C(27) (24-OH, 25-OH, and 27-OH, respectively). As 24-OH can exist in two epimeric forms (24(*S*)-hydroxycholesterol and 24(*R*)-hydroxycholesterol), for our investigations, we have chosen 24(*S*)-OH since it occurs naturally in this form.^24^ Additionally, synthetic 24(*R*)-OH is at least 1 order of magnitude less active compared to the natural isomer.^25^ Previous investigations involving oxysterols revealed that not only the kind of an additional polar group introduced into the cholesterol backbone^26,27^ but also its position in the sterane system or chain^28^ and molecule orientation^27,29^ strongly effect oxysterol organization at the phase boundary and modify the interactions with other lipids. This may directly impact the membranous activity of oxysterols as well as their other biological properties. The present work was aimed at getting a closer look at the surface behavior of sterols substituted in the isooctyl chain spread as Langmuir monolayers at the air/water interface.

## 2 Experimental and Theoretical Methods

### 2.1 Materials

24(*S*)-, 25-, and 27-hydroxycholesterol (abbr. 24-OH, 25-OH, and 27-OH) and cholesterol (Chol) were supplied by Avanti Polar Lipids. The compounds studied were of high purity (+99%) and were used as received. To prepare spreading solutions for monolayer experiments, chloroform of chromatographic grade (Sigma-Aldrich), stabilized with ethanol, was used. Ultrapure water (purified by a Millipore system) of 18.2 MΩ cm resistivity and a surface tension of 72.8 mN/m at 20 °C was used as a subphase.

### 2.2 Langmuir Monolayer Technique

The studied oxysterols were dissolved in chloroform (10^–3^ mol/dm^3^). Langmuir monolayers were prepared by dropping aliquots of the solutions with a Hamilton microsyringe (±2.5 μL) onto the surface of ultrapure water. The Langmuir film balance with a uniaxial compression NIMA 612D (double barrier, total area = 600 cm^2^) or a KSV 2000 (double barrier, total area 700 cm^2^) combined with a Brewster angle microscope was used to record the surface pressure (π)–area (*A*) isotherms. The surface pressure was measured with an accuracy of 0.1 mN/m using a Wilhelmy plate made from ashless chromatography paper (Whatman Chr1). Each isotherm was repeated three times to ensure that the curves were reproducible up to ±2 Å^2^/molecule. The subphase temperature was controlled by the use of a Julabo circulator. The monolayer stability was verified with the dynamic (compression–expansion cycles) and static (compression to a desired surface pressure and relaxation) methods. In order to characterize the mechanical properties of sterol monolayers, the surface compressional modulus (*C*_s_^–1^) values were calculated based on experimental π–*A* isotherms: .^30^*C*_s_^–1^ values below 25 mN/m suggest that the film is in a low-density liquid phase; the ranges of 25–50 and 100–250 mN/m are characteristic for liquid-expanded and liquid-condensed states, respectively, while for  above 500 mN/m, the film is in the solid state.^30^

Electric surface potential measurements were performed with the Kelvin probe (model KP2, NFT) mounted on a NIMA 612D trough. The vibrating plate was located ca. 1–2 mm above the water surface, while the reference electrode (platinum foil) was placed in the subphase. The surface potential measurements were reproducible to ±15 mV. The experimental values of the electric surface potential, Δ*V*, have been analyzed by applying the Helmholtz equation: Δ*V* = μ_⊥_/(*A*εε_0_),^30,31^ wherein μ_⊥_ denotes the vertical component of the dipole moment of the film molecule, ε_0_ is the vacuum permittivity, and ε is the permittivity of the monolayer. Because the value of ε is unknown, the changes of the effective dipole moment upon film compression are expressed by the apparent dipole moment, μ_a_ = μ_⊥_/ε.

### 2.3 Brewster Angle Microscopy

The BAM images of floating monolayers from the investigated compounds were registered using an ultraBAM (Accurion GmbH) installed over a KSV 2000 (double barrier, total area 700 cm^2^) Langmuir trough. The minimum reflection was set with a p-polarized laser beam (λ = 658 nm) incident on the pure aqueous surface at the Brewster angle (53.15°). The light reflected from the monolayer was collected through a 10× objective and lens system to a CCD camera. The BAM images presented herein show monolayer fragments of 720 μm × 400 μm.

### 2.4 PM-IRRAS

The PM-IRRAS spectra of Langmuir monolayers, compressed to the selected surface pressure values, were recorded with the KSV PM-IRRAS instrument at a fixed incidence angle of 76° and a minimum of 6000 scans for each shown spectrum (a spectral resolution of 8 cm^–1^). Measurements were performed at least twice to ensure the reproducibility of the results. The obtained spectra were processed with OMNIC software: background subtraction and smoothing (Savitzky–Golay method). Due to the fact that the literature lacks a precise analysis of molecular vibrations in 24-OH and 27-OH, the theoretical vibrational spectra were calculated using the density functional theory (DFT) method. The orientation of the transition dipole vector for each compound was predicted based on the analysis of the theoretical vibrational spectra calculated using the DFT method. The calculated normal modes were displayed using GaussView 6. The observation of the spatial visualization of the vibration allowed us to decide whether the main component of the vibration is perpendicular or parallel to the main axis of the molecule. This allowed us to assign bands appearing in the spectra to particular vibrations in the investigated molecules (Tables S1 and S2, Supporting Information).

### 2.5 Theoretical Calculations

Geometry optimization and vibrational spectra of 24-OH and 27-OH molecules were performed using DFT in the Gaussian 16 software package.^32^ All calculations were performed with the hybrid B3LYP functional^33,34^ and a basis set that includes diffuse and polarization functions, that is, 6-311++G(d,p).^35,36^ The dimer systems were calculated using additionally the D3 version of Grimme’s empirical dispersion with the original D3 damping function.^37^ The systems were optimized using the default UltraFine integration grid, default integral cutoffs, and a combination of EDIIS and CDIIS tight convergence procedures, without Fermi broadening. The base superposition error was eliminated by employing the counterpoise correction. The electron density and the Laplacian of the electron density in the bond critical points were calculated in the AIMAll software.^38^

Molecular dynamics simulations were performed using the Amber20 package.^39^ The simulated systems were built in Packmol software.^40^ Each system consisted of two rectangular symmetric monolayers, each monolayer having 128 lipid molecules (either 25-OH or 27-OH) separated by 30,000 water molecules. The partial atomic charge for 25-OH or 27-OH was calculated in Gaussian 16 using the Hartree–Fock method and the 6-31G(d) basis set. Periodic boundary conditions were utilized. The TIP3P model^41^ was used to simulate the water molecules. The energy of the systems was minimized by 50,000 steps. The systems were equilibrated by 75,000 steps with a 0.001 ps timestep, followed by 300,000 steps with a 0.002 ps timestep. Production calculations were carried out in an isothermal–isobaric ensemble with constant surface tension (*NP*γ*T*) with a 0.002 ps timestep. The simulation was carried out for 450 ns of the system evolution, and the last 10 ns were used for analysis. Hydrogen bonds were determined using geometric criteria: a distance between donor and acceptor heavy atoms less than 3.0 Å and an angle between donor, polar hydrogen, and acceptor at least 135°.

## 3 Results and Discussion

### 3.1 Surface Activity of 24-OH Is Strikingly Different Compared to 25-OH and 27-OH

**Figure 1:**
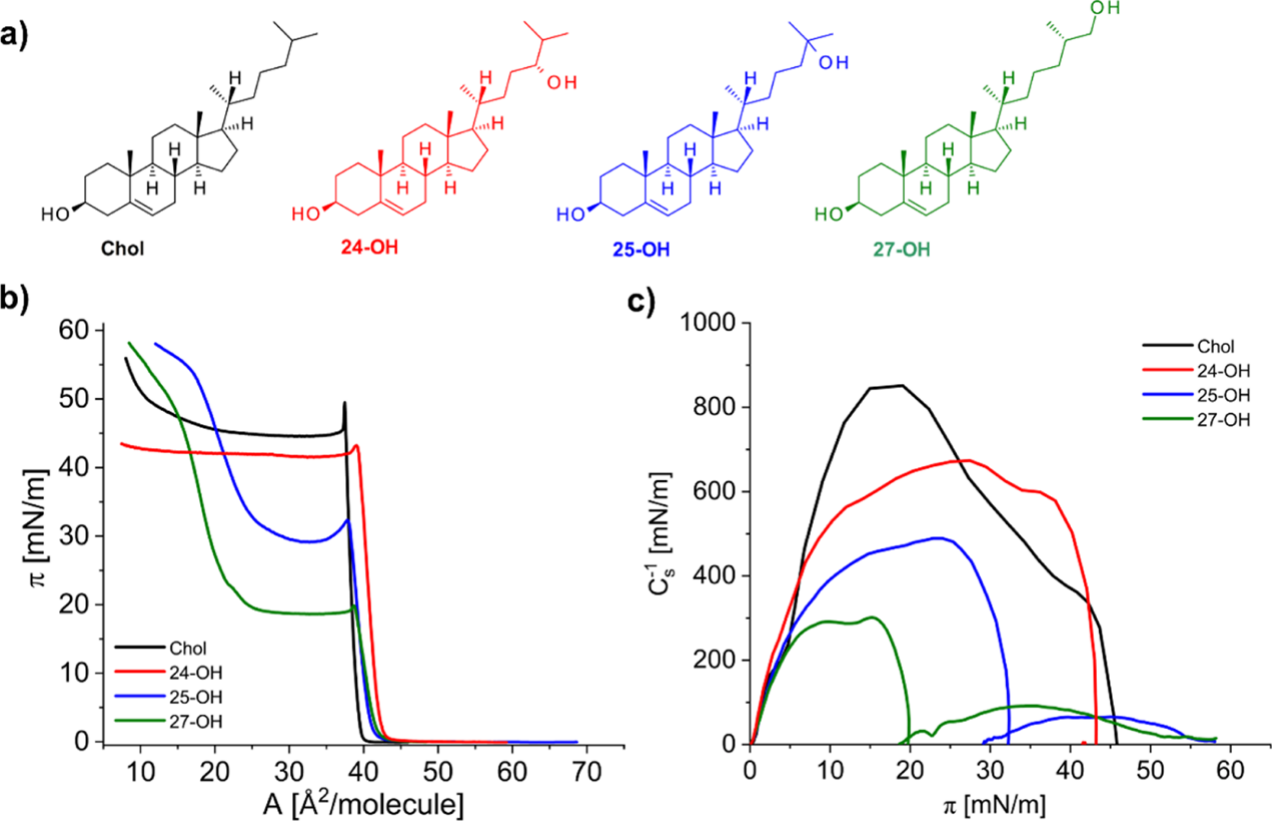
Molecular structures of cholesterol and its chain oxidized derivatives (a) together with surface pressure–area isotherms (b) and compressibility modulus dependencies (c) at 20 °C on the water subphase.

**Figure 2:**
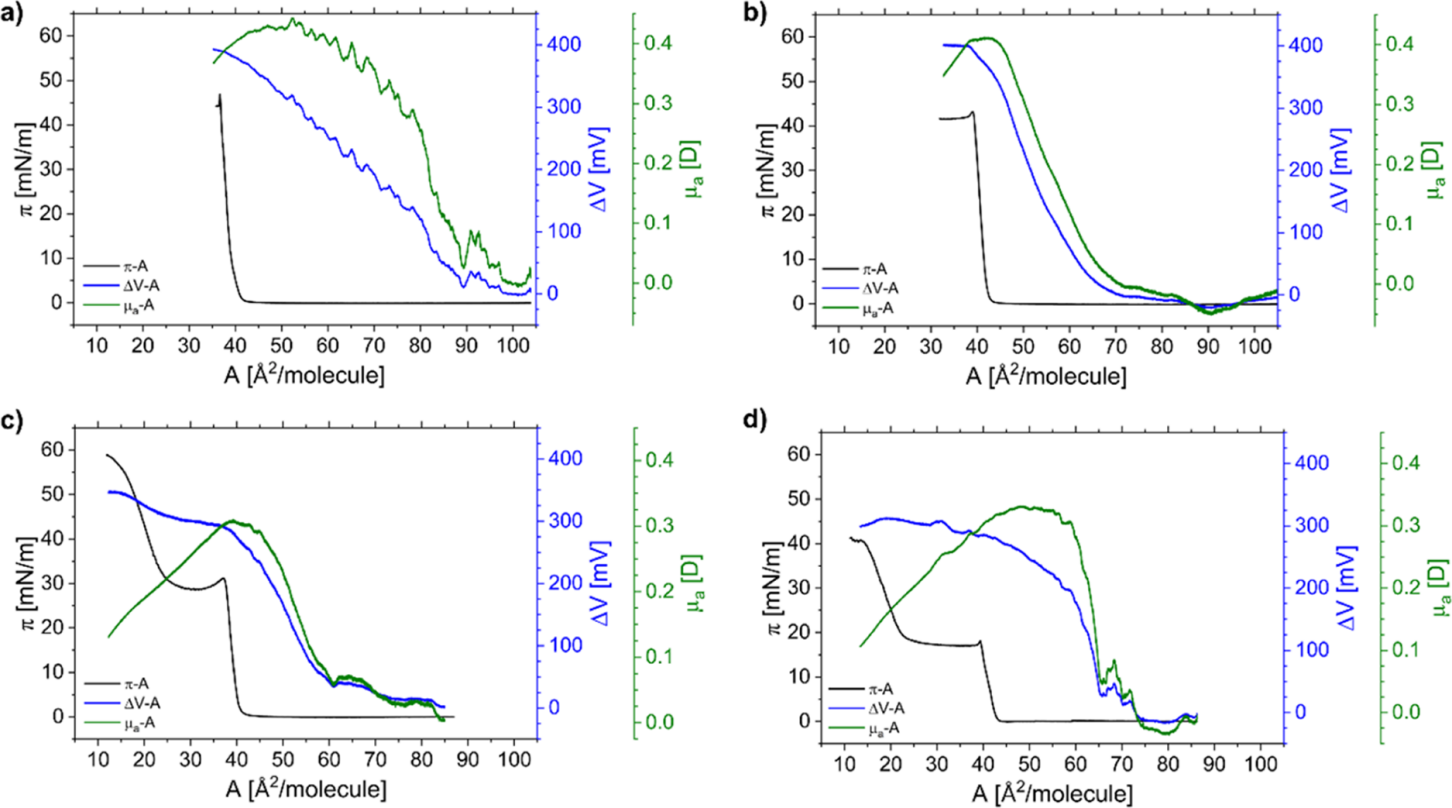
Surface pressure, surface potential, and apparent dipole moment versus area per molecule plots for monolayers from cholesterol (a), 24-OH (b), 25-OH^28^ (Copyright 2020 American Chemical Society) (c), and 27-OH (d) spread on water at 20 °C.

The structures of the physiologically crucial sterols oxidized in the octyl chain (24-OH, 25-OH, and 27-OH, Figure 1a) suggest that these compounds, from a physicochemical point of view, can be described as bipolar amphiphiles. As indicated in the Introduction section, such molecules may show unique surface anchoring and orientation at the boundary between the polar and the apolar medium. In the first step of our studies, we measured the π–*A* (Figure 1b) and Δ*V*–*A* (Figure 2) isotherms of the investigated oxysterols. Then, based on them, *C*_s_^–1^–*A* (Figure 1c) and μ_a_–*A* (Figure 2) dependences were calculated and plotted. The isotherms for cholesterol and 25-OH^28,42^ are shown for comparison purpose.

**Figure 3:**
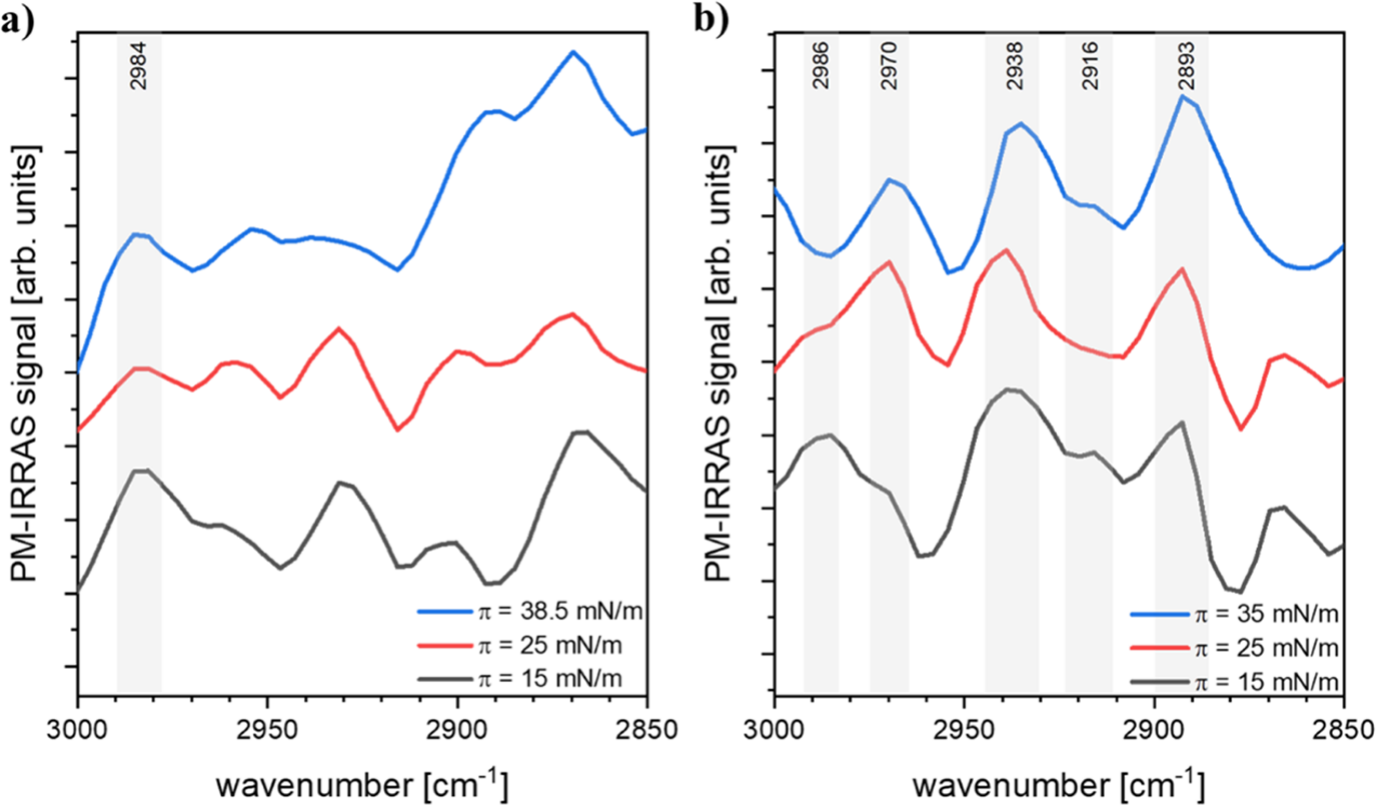
PM-IRRAS spectra in the hydrocarbon stretching vibration range registered at various surface pressure values for the 24-OH (a) and 27-OH (b) films spread on water at 20 °C.

The characteristics of the Langmuir monolayer from cholesterol are very well known (for detailed description, see ref (42) and references therein). This sterol forms highly reproducible and stable monolayers of condensed character at the air/water interface. The introduction of an additional hydroxyl group in all analyzed cases slightly shifts the lift-off area (the onset area of the surface pressure rise) toward higher values. For example, the 24-OH curve is shifted approximately 3 Å^2^/molecule toward the larger area per molecule values compared to the cholesterol curve. The 24-OH isotherm is also slightly less steep, and the collapse pressure (surface pressure corresponding to the discontinuity of the first derivative of π–*A*^43^) is lower (ca. 43 mN/m). Nonetheless, 24-OH, similarly to cholesterol, forms a condensed-type homogeneous monolayer, which is confirmed by compressibility modulus values (with maximum at ca. 675 mN/m), as well as the sequence of surface textures registered with the Brewster angle microscope. The BAM images (Figure S1, Supporting Information) show the coexistence of gas and liquid phases at low pressures and the homogeneity of the film until collapse, where 3D aggregates are formed. Further experiments have shown that the 24-OH isotherm is not influenced upon changing the barrier material or plate position (Figure S2, Supporting Information), and the monolayer is quite stable at the free water surface. Although in the static stability experiments (Figure S3, Supporting Information), an initial drop of π is observed in both low and higher regions of surface pressure, the film stabilizes over time. The electrical characteristics of the Langmuir monolayers from 24-OH and Chol are also similar (Figure 2a,b). The onset of electric surface potential change and the apparent dipole moment (the so-called critical area^44^) appear at about 80–90 Å. Both the surface potential and the apparent dipole moment rise simultaneously with surface pressure upon film compression, and for both analyzed compounds, they achieve the maximum values right before the collapse. All the above-mentioned results suggest that 24-OH, despite its bipolar structure, is anchored in the subphase with one hydroxyl group during compression, similar to cholesterol. To get a deeper insight into molecular orientation, conformation, and interactions^33^ upon compression of the 24-OH monolayer, we applied the PM-IRRAS method. Figure 3a shows the PM-IRRAS spectra in the hydrocarbon stretching vibration range.

As can be seen, the curves measured for 24-OH at surface pressures 15 and 25 mN/m are similar in the hydrocarbon stretching range in terms of band positions, which suggests lack of conformational changes during compression in the region below collapse. The decrease in the intensity of the CH_2_ asymmetric stretching band (at 2984 cm^–1^) upon compression indicates a slight change in the surface inclination of 24-OH. As proved by our molecular modeling studies, the transition moment of this band is parallel to the long axis of 24-OH. As a result, when the molecule becomes more vertical, the intensity of the band at 2984 cm^–1^ weakens. In turn, the bands in the spectrum of the collapsed film (at 38.5 mN/m) are significantly broadened and poorly defined, which is typical for this state. Regarding the fingerprint region where signals from vibrations of hydrophilic groups are visible, it is important to notice that compression does not significantly influence the position and intensity of these bands (Figure S4A, Supporting Information). This suggests that the surface anchoring and interactions of hydroxyl groups of 24-OH with the subphase remain unchanged upon compression below the collapse. A similar spectral behavior was described for cholesterol.^42^

The surface properties of 25-OH and 27-OH are quite different from those discussed above for Chol and 24-OH as it is visible in the shape of the pressure–area isotherms. Initially, π–*A* curves are parallel to the Chol isotherm; however, upon further compression, a characteristic pseudo-plateau region appears at surface pressure values ca. 30 and 20 mN/m for 25-OH and 27-OH, respectively (Figure 1b). Above this pseudo-plateau region, the isotherm rise continues, although with a lesser slope. Classical experiments based on the recording of surface pressure versus area per molecule confirmed that the 27-OH isotherm is not influenced by the barrier material (hydrophobic Teflon/hydrophilic Delrin) or the position of the Wilhelmy plate (Figure S2, Supporting Information). The static stability experiments (Figure S3, Supporting Information) prove the high stability of monolayers formed by 27-OH at surface pressure up to the pseudo-plateau region (up to ∼20 mN/m). For the monolayer compressed to higher pressures, above 30 mN/m, a sudden drop of surface pressure is first observed, and then the monolayer stabilizes at a pressure of 21 mN/m. The pre-plateau region is hardly influenced by temperature change within the range of 10–35 °C. However, the pressure of the plateau decreases with temperature rise (Figure S5, Supporting Information). In the case of 25-OH (analyzed in ref (28)), the molecular interpretation of the experimental Langmuir isotherm shows that 25-OH molecules are anchored to the surface with C(3)-OH or C(25)-OH. Such a unique arrangement explains the lower stability of 25-OH films compared to cholesterol. With the aid of complementary in situ surface-sensitive techniques (BAM, PM-IRRAS, and electrical surface potential change measurements), it was found that the kink in the isotherm (at ca. 30 mN/m) is due to the beginning of transition from mono- to bilayer structures. The analogical surface behavior is confirmed for 27-OH with the use of the above-mentioned techniques, which is briefly discussed below. First, the second surface pressure raise appears at approximately twice as much surface area as the lift-off point. Second, the texture visualization with the Brewster angle microscope (Figure S6, Supporting Information) shows the same transition sequence as previously observed for 25-OH. Namely, the typical gas–liquid coexistence at low surface pressures is visible, then the film becomes homogeneous until the pre-plateau region, where bright nuclei of bilayer structures start to appear and grow in number and size along the transition and with the subsequent pressure rise. Third, the electrical properties of 25-OH and 27-OH films are also analogous. Both surface potential and apparent dipole moment curves (Figure 2c,d) initially rise simultaneously with surface pressure upon film compression until the monolayer reaches the pressure of plateau. Starting from this point, the apparent dipole moment curve drastically decreases. This suggests that the dipole moments of molecules from the upper layer partly compensate those from the layer below. In consequence, depolarization of the surface film for both oxysterols (25-OH and 27-OH) occurs. Fourth, PM-IRRAS spectra for 27-OH also support the hypothesis that at the above surface pressure of 20 mN/m, the oxysterol molecules form bilayers on the surface of water (Figures 3b and S4B, Supporting Information). Generally, it can be noted that all spectra have well-defined bands, and the curves measured at 15 mN/m (pre-plateau region) are different from those probed at 25 and 35 mN/m (post-plateau region). Revealing the C-H stretching range (Figure 3b), it can be seen that the intensity of the bands appearing at low pressure (15 mN/m) at 2916 and 2986 cm^–1^ (CH_2_ symmetric stretching and CH_2_ asymmetric stretching, respectively) is increased compared to the spectra probed above the plateau. The transition moments of these particular vibrations are mainly parallel to the long axis of the 27-OH molecule; therefore, when 27-OH adopts a more vertical orientation at the water surface, these bands weaken or even become negative (the PM-IRRAS technique amplifies the signal from transition dipole moments parallel to the water surface^45^). In turn, the intensity of bands from CH_2_ stretching vibrations centered at ca. 2893 and 2970 cm^–1^ increases in the spectra probed at surface pressure values above the plateau (25 and 35 mN/m). As the transition dipole moments of these vibrations are mainly perpendicular to the molecule long axis, the increase of band intensity is related to (i) more vertical orientation of 27-OH at high pressures and (ii) an increase of molecular packing (in the sense of their number per unit area) associated with the formation of bilayers. The latter is confirmed by the ratio of the area under the deconvoluted band at ca. 2893 cm^–1^ in the spectra probed at 15 and 35 mN/m, which is approximately equal to 2 (Figure S7, Supporting Information). In the spectral region below 1500 cm^–1^, many significant bands from scissoring vibrations of C(3)-OH and C(27)-OH moieties appear (Figure S4B, Supporting Information). As can be seen, the compression of the film to the surface pressure above the plateau influences not only the intensity but also spectral positions of those bands (the exact changes in spectral positions and intensities are summarized in Table S2, Supporting Information). This suggests that the transition from mono- to bilayer structures leads to changes in molecular orientation of 27-OH molecules as well as affects the interactions of hydroxyl groups (probably at surface pressures above the plateau, the −OH groups are more often involved in the formation of hydrogen bonding with other oxysterol molecules rather than with water).

In summary, our investigations on the surface activity of biologically crucial sterols oxidized in the octyl chain showed a similarity of 24-OH to Chol, while the properties of 25-OH and 27-OH are strikingly different compared to the unoxidized compound. To understand how such a slight structural modification may lead to dramatic differences in surface properties, we employed theoretical modeling studies. In the first step of our theoretical investigations, the interaction energy for sterol molecules arranged in dimers with different mutual orientations was calculated (Table 1). The interaction energy values were calculated as Δ*E* = *E*_*xx*_ – 2*E*_*x*_, where *E*_*xx*_ is the total energy of the system consisting of two *xx* molecules (dimer) and *E*_*x*_ is the energy of one isolated X molecule.^46^ The following arrangements were analyzed: the octyl chains of both molecules directed to each other (0--- ---0), both molecules facing each other by the A-ring of the sterane system (---0 0---) and when the octyl chain of one molecule is facing the A-ring of the other (0--- 0---). In the Supporting Information, Figure S8, the optimized structures are shown, along with close-ups showing bond paths and Laplacian values of the electron density Δρ in bond critical points.

**Table 1:** Interaction Energies of Geometrically Converged 27-OH, 25-OH, 24-OH, and Cholesterol Dimers for Different Orientations

	corrected complexation energy (kcal/mol)			
arrangement	27-OH	25-OH	24-OH	Chol
0--- ---0	–8.18	–2.29	–1.65	–1.64
---0 0---	–9.27	–9.23	–9.27	–9.29
0--- 0---	–7.77	–8.01		

For all of the calculated systems, the strongest magnitude of interactions was found for molecules facing each other with the A-ring of the sterane system. The energy values are almost equal (approx. −9.3 kcal/mol), which can be expected for molecules with the same structure of the fused ring system. Analysis of the critical points of the bond shows that both oxygen atoms of the C(3)-OH groups are involved in hydrogen bonding. In addition, stabilizing intermolecular hydrogen–hydrogen interactions^47^ are present. Formation of a bilayer at the air–water interphase requires exposing hydrophilic groups from sterol molecules of at least one layer to the water. For cholesterol, this condition is met for dimers of type 0--- ---0 and 0--- 0---. As can be seen, the interaction energy value for the first system is low (value −1.6 kcal/mol, resulting from intermolecular hydrogen–hydrogen interactions), while the optimization for the second system was not convergent. This suggests that in the case of cholesterol, the formation of bilayers at the water surface is unfavorable. Analogous values of the interaction energy were obtained for 24-OH, which may be explained by the fact that the C(24)-OH group is masked by the hydrocarbon chain and cannot participate in the hydrogen bonding with another sterol molecule. On the contrary, the results obtained for 27-OH and 25-OH suggest that these molecules are capable of forming bilayers. First, dimers with the octyl chain of one molecule facing the A-ring of the other geometrically converged with favorable interaction energy values (−7.8 and −8.0 kcal/mol for 27-OH and 25-OH, respectively). These systems were stabilized by one hydrogen bond and additional intermolecular hydrogen–hydrogen interactions. Second, the 27-OH dimer composed of molecules with the A-ring exposed to the outside has the highest binding energy (−8.2 kcal/mol). Such a high interaction energy results from hydrogen bonds formed by both oxygen atoms of the C(27)-OH moiety and two additional stabilizing intermolecular hydrogen–hydrogen interactions. Interestingly, the results obtained from calculations employing empirical dispersion with the damping function allowed us to notice differences in the interaction energy for 25-OH, which was not observed in ref (28). Namely, the system of two 25-OH with the A-rings exposed to the outside is stabilized only by weak intermolecular hydrogen–hydrogen bonds, which results in a binding energy equal to −2.29 kcal/mol. These results show that the cholesterol and 24-OH systems with outwardly exposed polar groups are energetically unfavorable. The most energetically preferred arrangement for these molecules will prevent the formation of bilayers due to the hydrophobic interaction. On the other hand, the 25-OH and 27-OH systems, due to the hydrogen bonds formed by oxygen atoms from hydroxyl moieties at C(3) and C(25) or C(27), respectively, can easily form bilayers at the water/air boundary.

### 3.2 Differences in the Surface Activity of 27-OH and 25-OH

In the previous section, we highlighted the similarities in the surface activity of 25-OH and 27-OH. Now, we would like to emphasize the differences that may be crucial from the point of view of biological activity of these oxysterols. A very important issue to be discussed here is the reversibility of the monolayer to bilayer transition. Although the π-A characteristics recorded for 27-OH and 25-OH seem similar in the sense of the presence of the pseudo-plateau region, dynamic hysteresis experiments show significant differences. The dynamic compression–expansion cycles reveal no hysteresis both for films from 25-OH^28^ and 27-OH (Figure S9A, Supporting Information) compressed in the pre-plateau region. However, continuous multiple cycles performed at higher (post-plateau) pressure reveal—in the case of 27-OH—a visible small hysteresis of isotherms for the consecutive compression–expansion cycles (Figure S9B, Supporting Information). The isotherm shape remains the same, while a systematic decrease in the area per molecule suggests a slight loss of molecules from the surface. The BAM images revealed that in the texture characteristic for gas/liquid coexistence, small fragments of bilayer structures remain present during the beginning of the second and third compression–expansion cycle (Figure S9C, Supporting Information). This suggests that the bilayer formation process is to some extent (but not fully) reversible in the case of 27-OH. In contrast, hysteresis experiments for 25-OH together with BAM microscopy indicated complete irreversibility of bilayer formation.^28^

Based on hysteresis experiments, it is possible to calculate the free energy of the compression/expansion cycle (Δ*G*^comp/exp^), similarly to the thermodynamic functions of mixing^48^
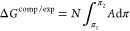
1where *N* is the Avogadro number. In our experiments, the integral was calculated between π_1_ = 1.2 mN/m and π_2_ = 39.8 mN/m. The free energy of hysteresis can be quantified by a difference between the free energy of compression and expansion^48−51^
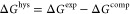
2

Other thermodynamic functions for hysteresis can be obtained from the following equations
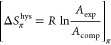
3
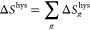
4
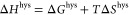
5

Table 2 compiles the obtained results for 27-OH in comparison with 25-OH.

**Table 2:** Thermodynamic Functions of Hysteresis: The Free Energy of Hysteresis (Δ*G*^hys^), the Entropy of Hysteresis (Δ*S*^hys^), and the Enthalpy of Hysteresis (Δ*H*^hys^) for Chain-Oxidized Oxysterols 27-OH and 25-OH

oxysterol	cycle	Δ*G*^hys^ [kJ/mol]	Δ*S*^hys^ [J/mol K]	Δ*H*^hys^ [kJ/mol]
27-OH	first	–1.83	–2.76	–2.64
	second	–1.96	–3.12	–2.87
	third	–2.00	–3.51	–3.03
25-OH	first	–2.85	–3.70	–3.94
	second	–0.45	–0.74	–0.67
	third	–0.42	–0.70	–0.63

The calculated thermodynamic hysteresis functions are different from zero and have negative values. This proves that we are dealing with not ideal but real systems, where some amount of energy (Δ*G*^hys^) accumulates during compression and is not fully returned upon expansion.^48−50^ This occurs when various molecular arrangements of different cohesive and viscoelastic properties are formed during the cycle. The negative values of Δ*S*^hys^ indicate the formation of entropically unfavorable, ordered and closely packed molecular structures, which interact with enthalpically favorable (exothermic) interactions (negative Δ*H*^hys^ values). Such arrangements can arise upon film compression, when the hydrogen-bonded network breaks, leading to the formation of aggregates, which may not be fully reversed on expansion.^51^ It is intriguing that in the case of 27-OH, these arrangements undergo a slow reorganization, while those formed by 25-OH behave irreversibly. To understand this phenomenon, we performed molecular dynamics studies, which are presented later on.

**Figure 4:**
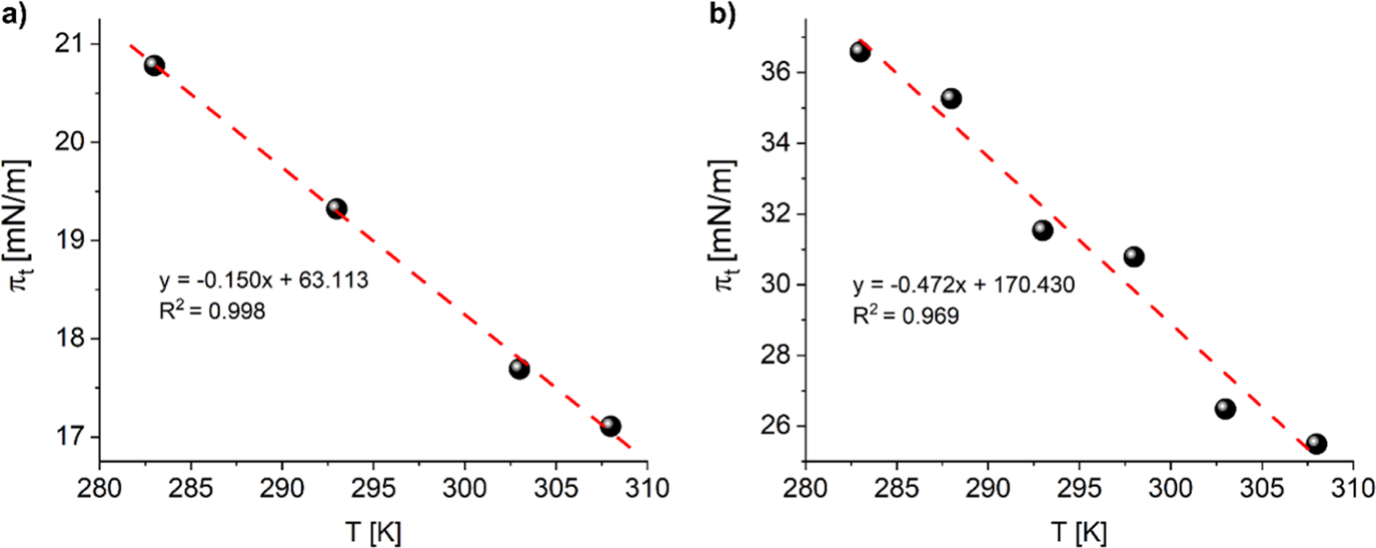
Dependence of the surface pressure value of the middle of the plateau region (π_*t*_) on the temperature for 27-OH (a) and 25-OH (b).

A further insight into the monolayer to bilayer transition can be obtained by analyzing the effect of temperature on the plateau transition. Based on the measured π-A isotherms in the range of 10–35 °C (Figure S5, Supporting Information for 27-OH, data for 25-OH taken from ref (28)), the following values were read and are collected in Table 3: *A*_b_ and *A*_e_ (area per molecule at the beginning and end of the plateau, respectively) and π_b_ and π_e_ (surface pressure at the beginning and end of the plateau, respectively). Then, the dependencies of the surface pressure value of the middle of the plateau region (π_*t*_ = (π_b_ + π_e_)/2) on the temperature were plotted for 27-OH and 25-OH (Figure 4).

**Table 3:** Δ*H*_*t*_ and Δ*S*_*t*_ Values for the Plateau Transition of 25-OH and 27-OH

oxysterol	*T* [K]	*A*_b_ [Å^2^/molecule]	*A*_e_ [Å^2^/molecule]	π_b_ [mN/m]	π_e_ [mN/m]	π_*t*_ [mN/m]	dπ_*t*_/d*T* [mN/m K]	dγ/d*T* [mN/m K]	Δ*H*_*t*_ [kJ/mol]	Δ*S*_*t*_ [J/mol K]
25-OH	283	33.85	25.34	36.42	36.75	36.59	–0.472	–0.153	4.63	16.3
	288	33.99	26.06	34.91	35.61	35.26			4.39	15.2
	293	34.76	30.14	31.53	31.53	31.53			2.60	8.9
	298	35.41	31.88	30.61	30.96	30.79			2.02	6.8
	303	37.14	33.96	26.37	26.59	26.48			1.85	6.1
	308	37.36	35.29	25.36	25.62	25.49			1.22	4.0
27-OH	283	37.94	22.23	20.24	21.32	20.78	–0.150		–0.08	–0.3
	293	38.01	25.41	19.06	19.58	19.32			–0.07	–0.2
	303	39.11	25.33	17.34	18.04	17.69			–0.07	–0.2
	308	39.75	26.94	16.85	17.36	17.11			–0.07	–0.2

As can be observed, the influence of temperature on π_*t*_ is more significant for 25-OH, while for 27-OH, the effect is smaller (the variances in π_*t*_ in the same temperature range are almost 3 times smaller for 27-OH versus 25-OH). Based on the slope of the dependencies in Figure 4 (dπ_*t*_/d*T*) and the data read from isotherms, the thermodynamic functions for the plateau transition can be calculated. Changes in enthalpy Δ*H*_*t*_ for the transition at constant temperature and pressure are described by the adapted Clausius–Clapeyron equation^52,53^
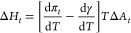
6where Δ*A*_*t*_ = *A*_e_ – *A*_b_ and dπ_*t*_/d*T* is the slope of the dependence π_*t*_ (T) in Figure 4. The value of dγ/d*T* (for temperatures between 10 and 35 °C), where γ is the surface tension of water, is equal to −0.153 mN m^–1^ K^–1^.^54^

The obtained values of Δ*H*_*t*_ and Δ*S*_*t*_ are summarized in Table 3 and point to significant differences in the thermodynamics of bilayer formation between 25-OH and 27-OH.

In the case of 25-OH, the phase-transition enthalpy values are positive (ca. 1.22–4.63 kJ/mol), which suggests that the formation of bilayers from 25-OH is endothermic. The irreversibility of the transition is confirmed with positive values of entropy. In contrast, for 27-OH, both Δ*H*_*t*_ and Δ*S*_*t*_ values are close to zero, which indicates in this case the reversibility of the monolayer–bilayer transition.

For information on the molecular origin of the observed differences in the transition behavior in 25-OH versus 27-OH, we applied molecular dynamics simulations. Since the exact reproduction of hysteresis experiments in molecular dynamics calculations is not possible, therefore, we decided to apply the simplified approach. Systems of two monolayers, each consisting of 128 molecules with random orientation, were simulated for a surface pressure of 35 mN/m as well as lower values of 24 and 15 mN/m. We let these systems evolve for 450 ns, and the last 10 ns were used for analysis. We were interested in differences between the behavior of bilayers composed of 25-OH or 27-OH. For a pressure of 35 mN/m, both systems were ordered quickly. After 20 ns, the molecules were aligned in parallel, and the area per molecule did not change. For the last 10 ns of system evolution, the average area per lipid was equal to 36.6 and 38.0 Å^2^, while the tilt angles (the angle between the longitudinal axis of the molecule and the normal to the surface) were 6.5 and 24.4° for 27-OH and 25-OH, respectively (see Figure S10, Supporting Information). The population of molecules anchored in water with the C(3)-OH group was in a slight predominance (55.3 and 54.4% of the molecules were anchored in the water layer with C(3)-OH for the 27-OH and 25-OH system, respectively; see the electron density functions in Figure S11, Supporting Information). At the same time, the hydration of the C(3)-OH groups was greater than that of the additional hydroxyl groups (see the radial distribution functions in Figure S12, Supporting Information). Analysis of the potential energy values of the systems reveals significant differences in the intermolecular interactions. We found a higher value of the potential energy of the van der Waals interaction for the 27-OH system (*E*_vdW_ = −6546 kJ/mol, while for 25-OH *E*_vdW_ = −6060 kJ/mol). An even greater difference was apparent for the potential energy of the noncovalent electrostatic interactions. For the 27-OH system, the energy *E*_elec_ = −2024 kJ/mol, while for the 25-OH, *E*_elec_ = 6782 kJ/mol. These values allowed us to conclude that there are more attractive intermolecular interactions for the 27-OH system than for the 25-OH system.

In the next step, we examined the hydrogen bonds formed between oxysterol molecules. We found that for the last 10 ns of the system evolution, an average of 89.77 hydrogen bonds were present in the 27-OH system, while for the 25-OH system, 72.43. For a more in-depth study of hydrogen bonds, we chose those that were present for at least 80% of the analyzed time of system evolution. The complexes formed by these bonds are presented in the Supporting Information. Eleven bonds for 27-OH were formed between the layers and 12 for 25-OH (see Figure S13, Supporting Information). Interesting differences appear for the systems with a surface pressure of 24 mN/m. In this case, the 27-OH system was arranged in 20 ns from the initial bilayer to a stable monolayer, for which *E*_vdW_ = −5695 kJ/mol and *E*_elec_ = −1336 kJ/mol. In the case of 25-OH, the layered structure was disturbed, and an unstable system was created, similar to the one described in our previous paper.^28^ The formation of a stable monolayer by the 27-OH system under reduced pressure can be explained by the large number of hydrogen bonds formed in the system of these molecules and the strong attractive electrostatic and van der Waals interactions, which is reflected in the high energy values of the 27-OH system, larger than those for 25-OH. This, in turn, may explain the different behavior of the investigated oxysterols after decompression of the bilayer system. 27-OH forms a monolayer, while 25-OH remains in a 3D arrangement. Eventually, for the lowest surface pressure, 15 mN/m, both systems were thermodynamically unstable and the layered structure was disturbed.

## 4 Conclusions

The introduction of an additional polar group into the cholesterol backbone modifies the properties of molecules (such as polarity and hydrophilicity) and makes oxysterols an extremely interesting group of compounds with a wide range of activities. This seemingly minor structural change has significant consequences, especially in the membrane environment, where the molecular arrangement plays a key role. Nevertheless, oxysterols, depending on the type and position of the polar group, may affect membrane structure, fluidity, permeability, and functioning. So far, most research has concerned the differences between ring- and chain-oxidized sterols, while in this paper, we focused on the surface activity of oxysterols with different positions of the additional hydroxyl group in the side chain (C(24), C(25), and C(27)) using the Langmuir monolayer approach. The results from our studies showed some interesting consequences of the introduction of a hydroxyl moiety into the isooctyl chain of cholesterol. Namely, when the polar group is located at the end of the side chain (at C(27)), the resulting bipolar molecule is vertically oriented in the surface layer and is anchored in the polar phase with the C(3)-OH or C(27)-OH group. The dehydrated polar groups of 27-OH molecules interact with each other via van der Waals and hydrogen bonding. These interactions may occur in parallel (between molecules arranged in the same monolayer) and vertically (between molecules located in adjacent layers). This explains the observed ability of 27-OH molecules to undergo a reversible transition from mono- to bilayer structure upon compression at the water–air interface and a distinct membrane translocation mechanism.^55^ Shifting the polar group to C(25) causes slight modifications in the surface activity of 25-OH compared to that of 27-OH. Namely, a higher surface pressure is required to occur the phase transition from the mono- to bilayer structure, and this process becomes irreversible. This can be explained by geometric considerations (25-OH molecules are more inclined in the layer, and therefore, upon decompression, the reconstruction of the monolayer becomes more difficult). Moreover, the C(25)-OH group is shielded by two methyl moieties, and therefore, its conformational lability is reduced. When the hydroxyl group is introduced at C(24), the surface properties change dramatically. The C(24)-OH moiety becomes unable to form hydrogen bonds with water as well as with other 24-OH molecules due to the steric hindrance of the isopropyl moiety. As a result, 24-OH behaves similarly to cholesterol and is capable of forming stable rigid monolayers at the water surface, which are unable to transition to bilayer structures.

The described differences in the ability of individual laterally oxidized sterols to self-organize at the phase boundary may result in their specific biological properties. Indeed, 25-OH and 27-OH, whose surface activity is quite similar, show alike behavior, for example, they are able to block SARS-CoV-2 infection, although by different mechanisms.^56,57^ On the other hand, 24-OH, which has different surface characteristics, does not show such properties.^58^ We hypothesize that the ability to form hydrogen bonds may be responsible for the observed distinct physiological activity of 25-OH and 27-OH compared to 24-OH. A similar suggestion, indicating the changes in the local network of hydrogen bonds, has been made to interpret the differences in biological activity for the cis and trans isomers of ceramides.^59^

## Supporting Information Available

Surface pressure–area isotherms, BAM images, PM-IRRAS spectra, bond paths for dimers, distributions of area per molecule, tilt angle, electron density from MD, snapshots of hydrogen-bonded complexes, and assignments of PM-IRRAS bands (PDF)
