# Supplementary material for: Site of the Hydroxyl Group Determines the Surface Behavior of Bipolar Chain-Oxidized Cholesterol Derivatives—Langmuir Monolayer Studies Supplemented with Theoretical Calculations
Source: J Phys Chem B. 2023 Feb 23;127(9):2011–21. doi: 10.1021/acs.jpcb.2c08629 (PMC10009745; doi:10.1021/acs.jpcb.2c08629)
Supplement: Supplementary file 1 — jp2c08629_si_001.pdf [file jp2c08629_si_001.pdf]

Supporting Information

for

The Site of the Hydroxyl Group Determines Surface Behavior of Bipolar Chain-Oxidized Cholesterol Derivatives – Langmuir Monolayer Studies Supplemented with Theoretical Calculations

Anna Chachaj–Brekiesz[1,*], Anita Wnętrzak[1], Jan Kobierski[2],
Aneta D. Petelska[3], Patrycja Dynarowicz–Latka[1]

1) Faculty of Chemistry, Jagiellonian University, Gronostajowa 2, 30–387 Kraków, Poland

2) Department of Pharmaceutical Biophysics, Faculty of Pharmacy, Jagiellonian University Medical College, Medyczna 9, 30–688 Kraków, Poland

3) Faculty of Chemistry, University of Bialystok, Ciolkowskiego 1K, 15–425 Bialystok, Poland

*) corresponding author, e-mail: anna.chachaj@uj.edu.pl

A)

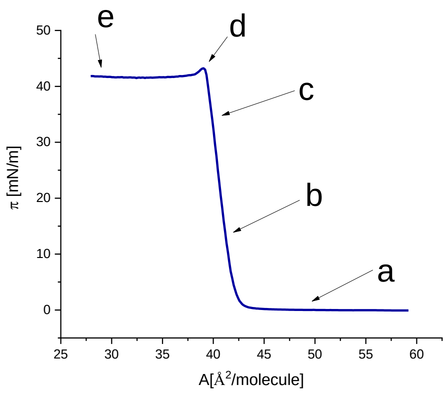

B)

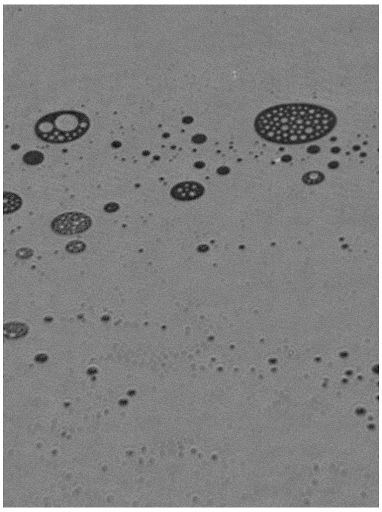

a) π = 0.01 mN/m

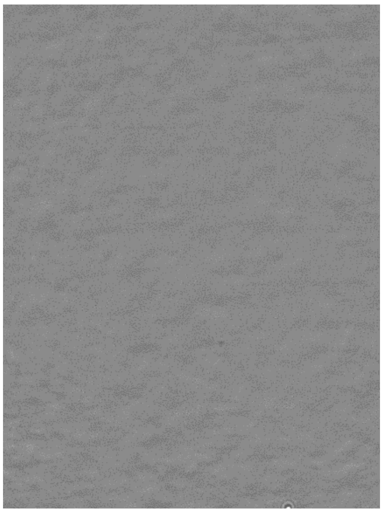

b) π = 10 mN/m

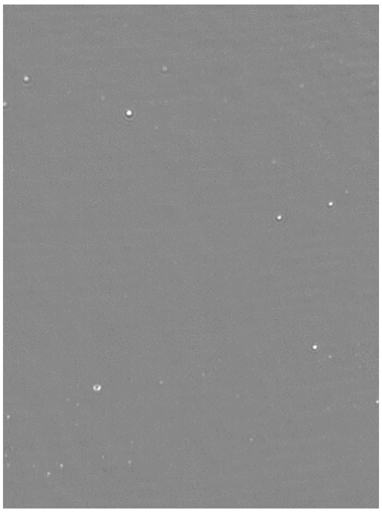

c) π = 30 mN/m

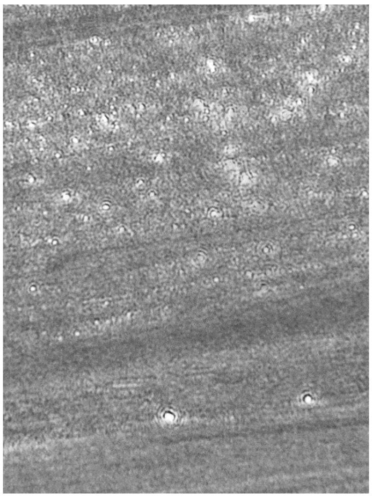

d) π = 42.0 mN/m

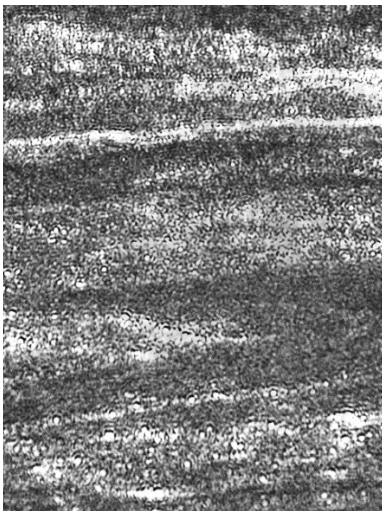

e) π = 41 (after collapse)

Figure S1. Surface pressure-area isotherm for 24-OH monolayer spread on water at 20 °C (A). Textures of 24-OH films on water subphase registered with BAM at different values of surface pressure. The isotherm regions corresponding to each texture are marked with corresponding letters (a-h) (B).

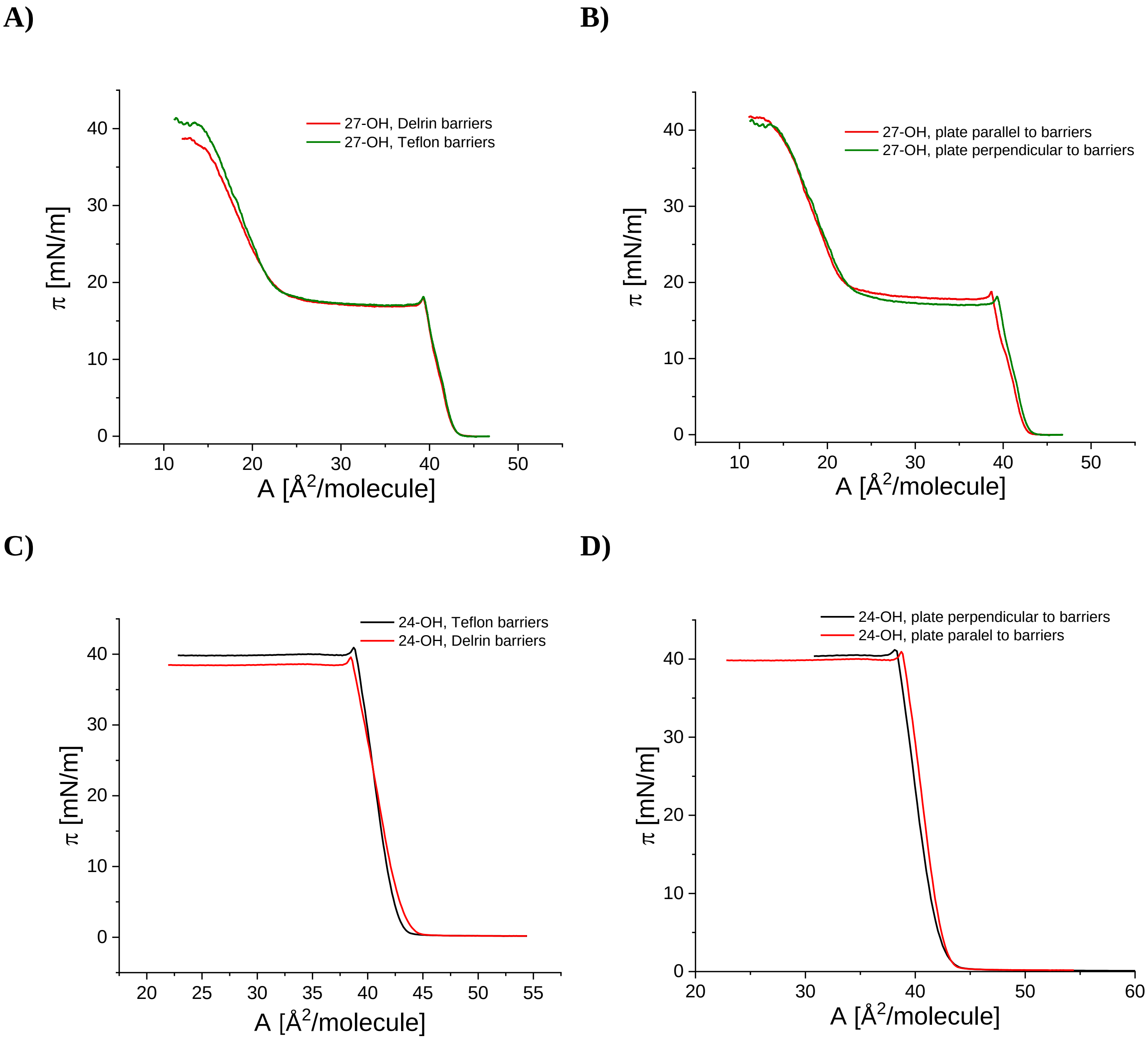

Figure S2. Influence of (A, C) the barrier material (Delrin *vs* Teflon) and (B, D) the orientation of Wilhelmy plate in respect to barriers position on surface pressure-area isotherms of 27-OH (A, B) and 24-OH (C, D) (recorded at 20 °C).

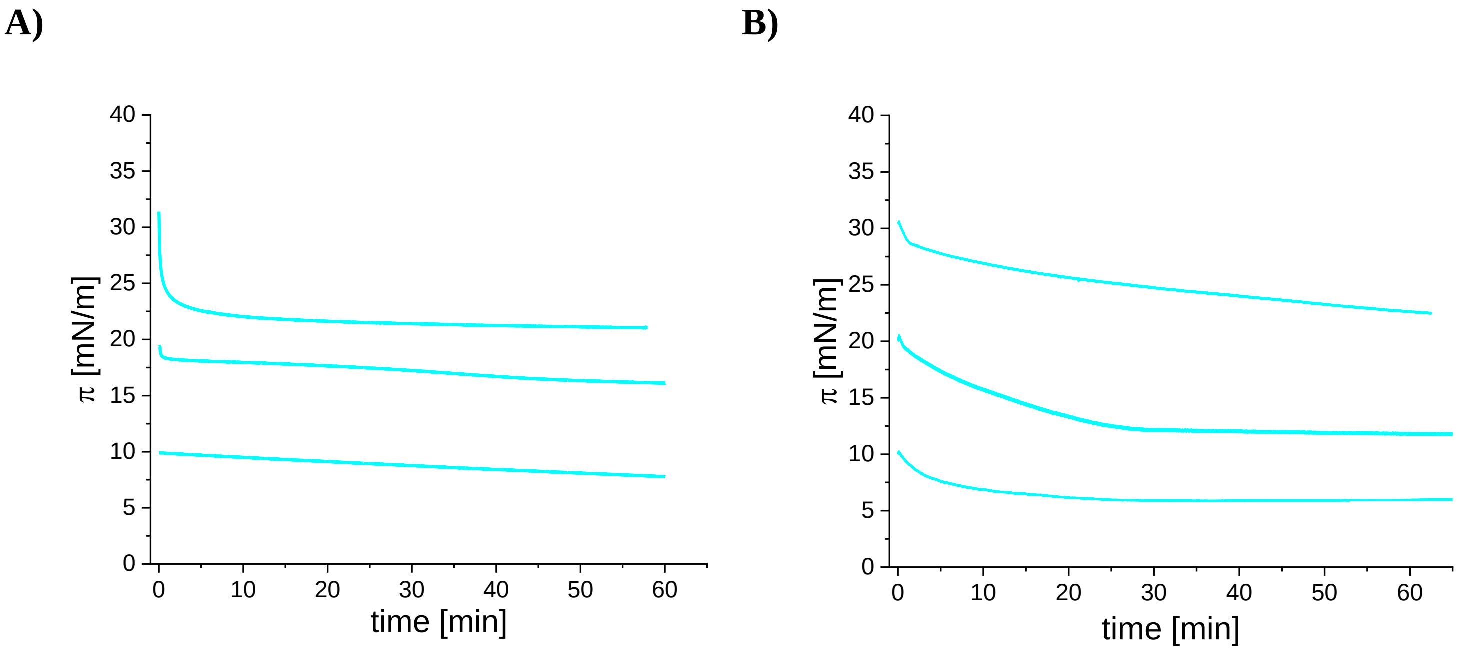

Figure S3. Static stability experiments of Langmuir monolayers from 27-OH (A) and 24-OH (B) at water (recorded at 20 °C).

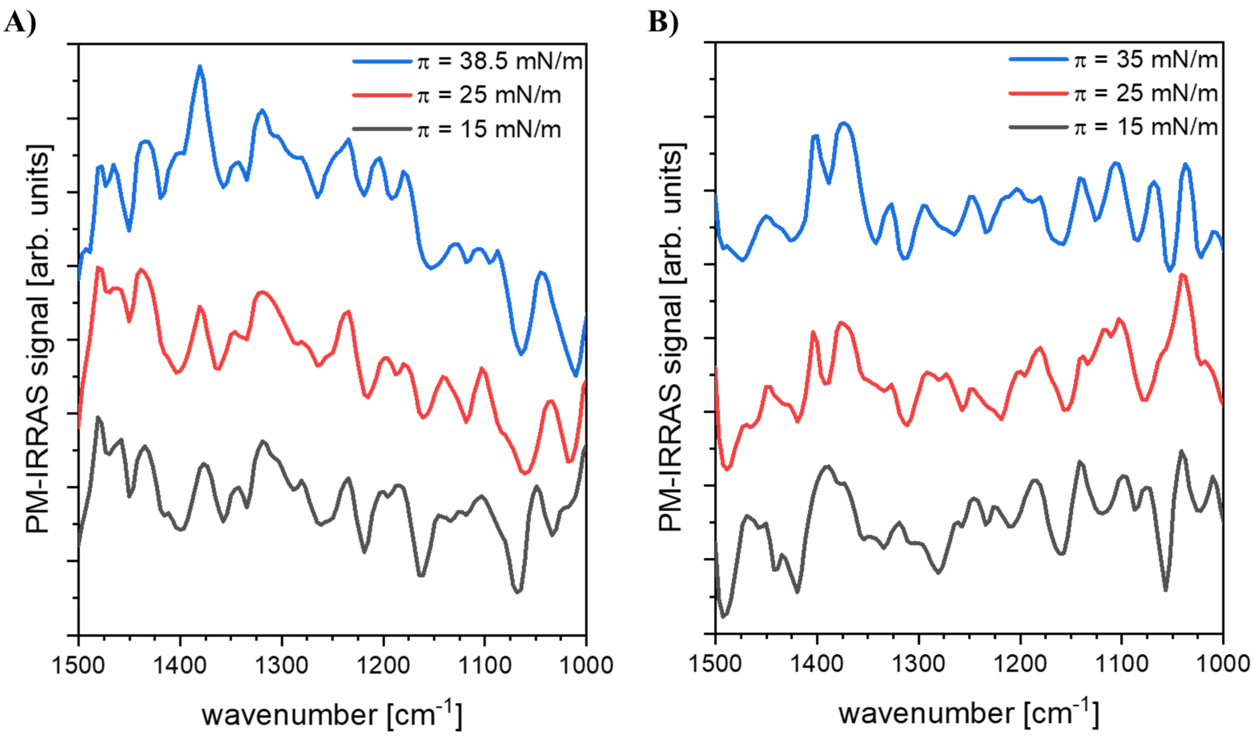

Figure S4. PM-IRRAS spectra in the 1500-1000 cm^{-1} range registered for the 24-OH (A) and 27-OH (B) films spread on water at 20 °C.

Table S1. Assignment of main vibrational modes observed in PM-IRRAS spectra of 24-OH.

wavenumber [cm^{-1}]				description of the vibration*
scaled calculated spectrum	PM-IRRAS spectrum			
	π = 15 mN/m	π = 25 mN/m	π = 38.5 mN/m	
3011	2984	2983	2983	CH_2 asymmetric stretching (‖)
3001	2960	2958	2953	CH_2 asymmetric stretching in tail (‖ and ⊥)
2921	2928	2930	broad	CH_2 symmetric stretching (‖ and ⊥)
2877	2901	2900	2892	CH_2 symmetric stretching (‖ and ⊥)
2838	2867	2870	2869	CH stretching (⊥)
1476	1480	1480	1480	CH_2 scissoring (‖ and ⊥)
1463	1459	1460	1465	CH_2 scissoring (‖)
1434	1435	1437	1435	CH_2 scissoring (⊥)
1380	1377	1379	1381	CH_3 scissoring (⊥)
1356	1342	1346	1344	CH_3 scissoring (‖ and ⊥)
1325	1318	1318	1320	C(24)-OH scissoring (⊥)
1282	1279	1279	1279	C(3)-OH scissoring (‖), CH_2 wagging (‖)
1258	1235	1235	1235	CH_2 wagging in chain (‖)
1200	1201	1198	1205	C(3)-OH scissoring (‖)
1170	1182	1177	1177	ring deformation, CH_2 twisting (‖)
1120	1143	1140	1130	CH_2 twisting (‖)
1086	1102	1102	1104	C(24)-OH scissoring (⊥)
1043	1049	1037	1043	C(3)-O stretching (‖)

* (‖) or (⊥) symbols refer to the predicted orientation of the dipole moment vector with respect to the long axis of the 24-OH molecule.

Table S2. Assignment of main vibrational modes observed in PM-IRRAS spectra of 27-OH.

wavenumber [cm^{-1}]				description of the vibration*
scaled calculated spectrum	PM-IRRAS spectrum			
	π = 15 mN/m	π = 25 mN/m	π = 35 mN/m	
3009	2986	2988	-	CH_2 asymmetric stretching (ǁ and ⊥)
2975	2970	2970	2969	CH_2 and CH stretching (⊥)
2939	2938	2939	2935	CH_2 symmetric stretching (ǁ and ⊥)
2927	2916	-	2916	CH_2 symmetric stretching (ǁ and ⊥)
2893	2893	2892	2891	CH_2 symmetric stretching (⊥)
2861	2867	2867	-	CH stretching (⊥)
1465	1467	1467	-	CH_2 scissoring (ǁ)
1436	1448	1448	1448	CH_2 scissoring (ǁ and ⊥)
	1390	1401	1401	CH_3 scissoring (⊥)
	1373	1375	1373	CH_3 scissoring (⊥)
1400	1319	1312 (negative)	1315 (negative)	C(27)-OH scissoring (ǁ and ⊥)
1391	1296	1291	1293	C(3)-OH scissoring (ǁ)
1262	1278 (negative)	1274	1270 (negative)	C(3)-OH scissoring (ǁ)
1252	1246	1248	1248	C(3)-OH and C(27)-OH scissoring (ǁ)
1236	1224	1222 (negative)	1218	C(27)-OH scissoring (ǁ)
1204	1207 (negative)	1203	1203	C(27)-OH scissoring (ǁ)
1192	1186	1181	1181	ring deformation, CH_2 twisting (ǁ and ⊥)
1137	1138	1138	1138	CH_2 twisting (ǁ)
1120	1098	1102	1106	C(3)-O stretching, C(3)-OH scissoring (ǁ)
1052	1074	1076 (negative)	1070 or 1083 (negative)	C-O-H twisting (ǁ)
1036	1040	1040	1038	C-O stretching (ǁ)
993	1010	1010	1010	rings deformation and C-O stretching

* (ǁ) or (⊥) symbols refer to the predicted orientation of the dipole moment vector with respect to the long axis of the 27-OH molecule.

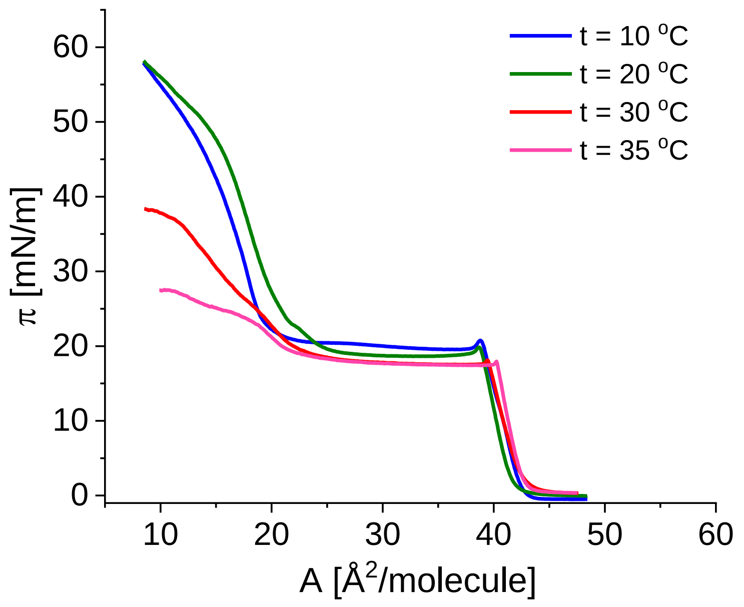

Figure S5. Influence of the temperature of subphase on surface pressure-area isotherms of 27-OH.

A)

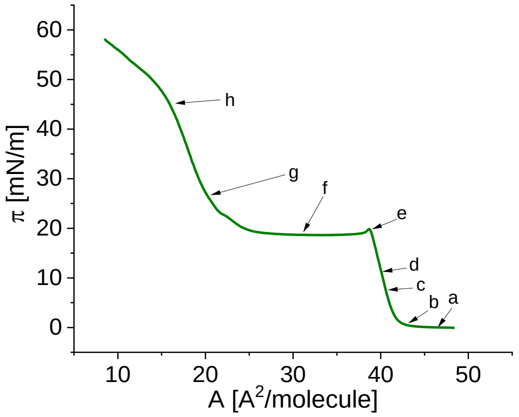

B)

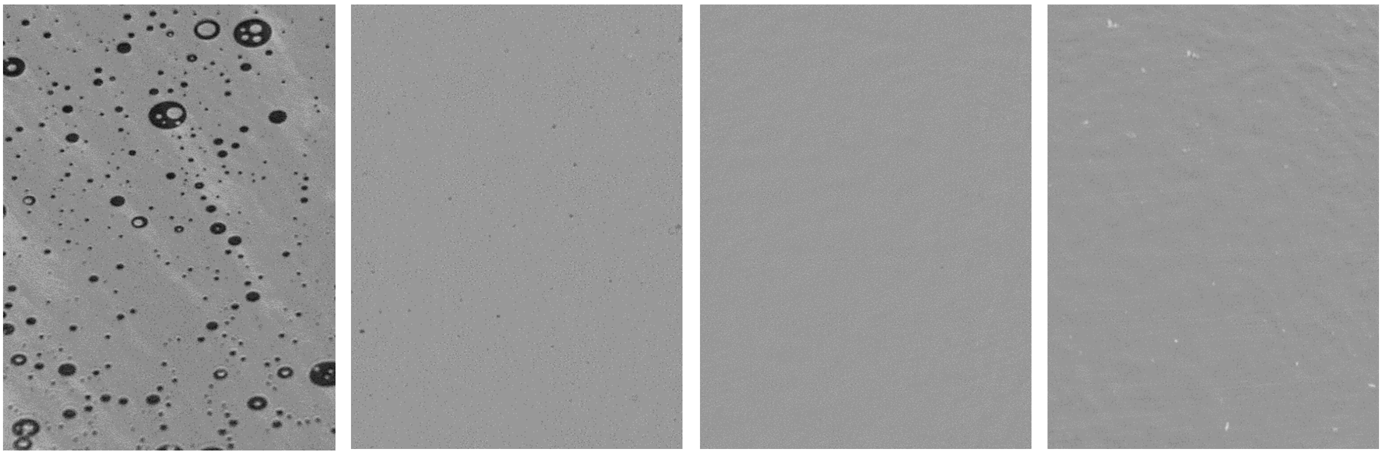

a) π = 0.01 mN/m b) π = 0.35 mN/m c) π = 8.0 mN/m d) π = 13.0 mN/m

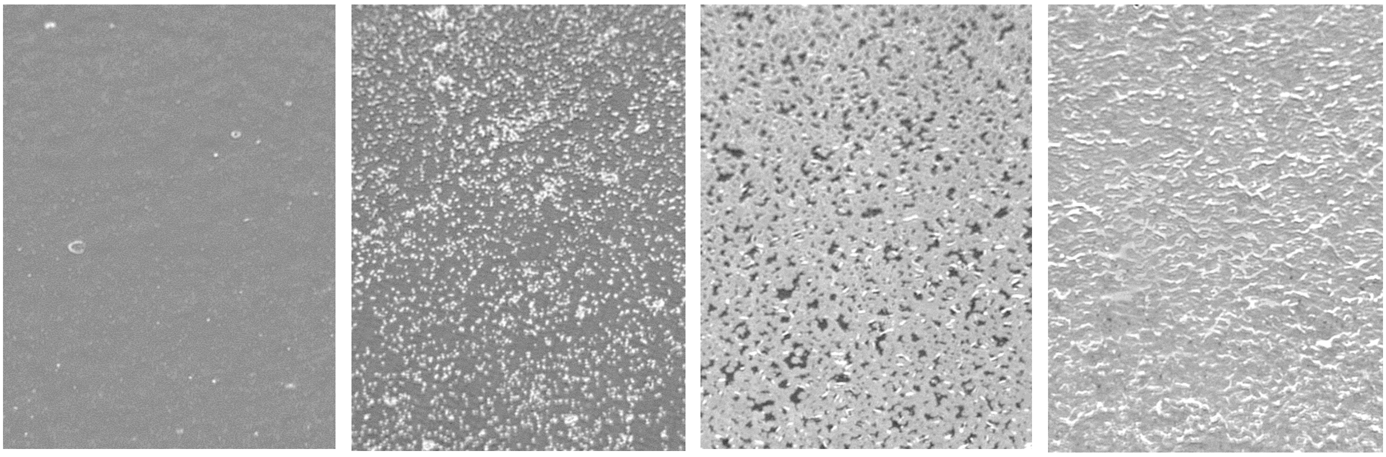

e) π = 20.0 mN/m f) „plateu” g) π = 25.0 mN/m h) π = 40.0 mN/m

Figure S6. Surface pressure-area isotherm for 27-OH monolayer spread on water at 20 °C (A). Textures of 27-OH films on water subphase registered with BAM at different values of surface pressure. The isotherm regions corresponding to each texture are marked with corresponding letters (a-h) (B).

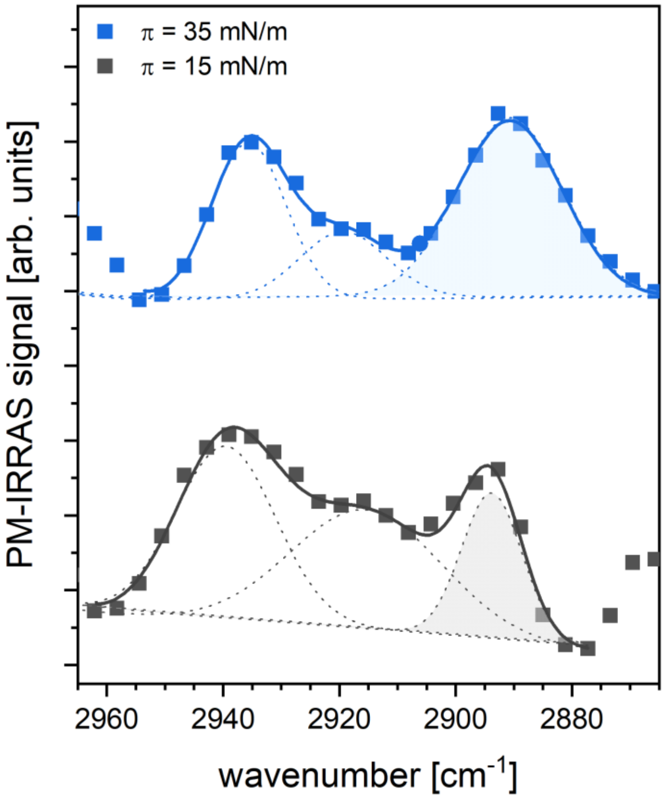

Figure S7. Deconvolution CH_2 stretching vibrations centered band (at c.a. 2893 cm^{-1}) in PM-IRRAS spectra of 27-OH. The ratio of area under deconvoluted band is approximately equal to 2.

A) 27-OH, 0--- ---0:

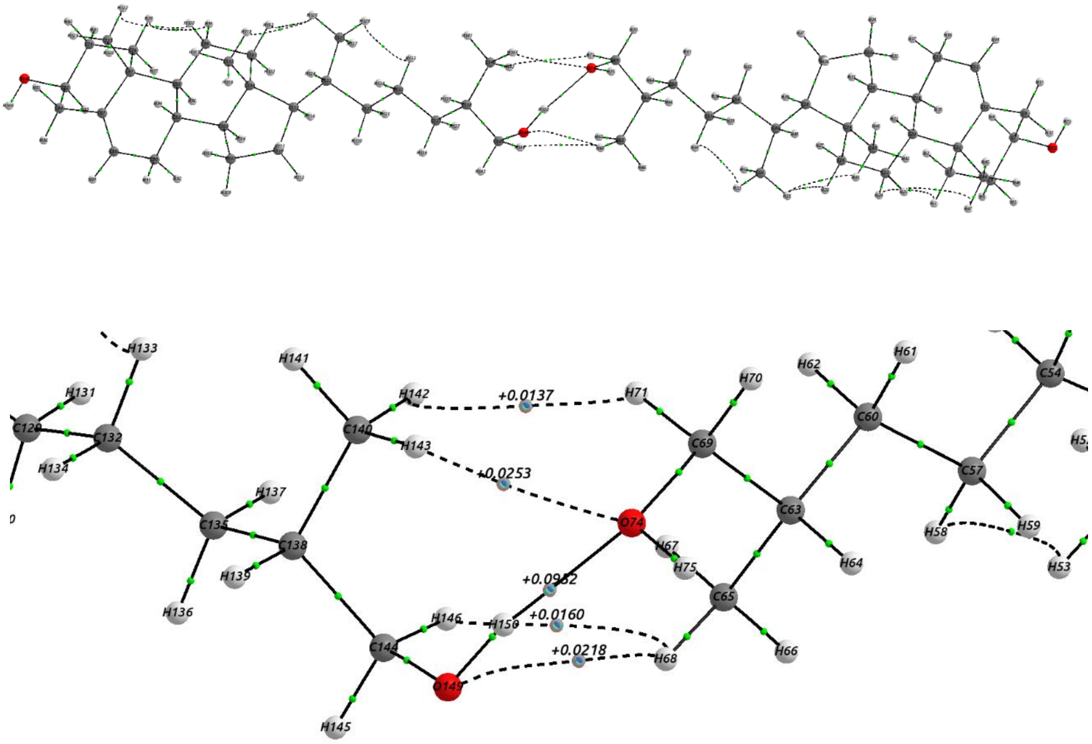

27-OH, ---0 0---:

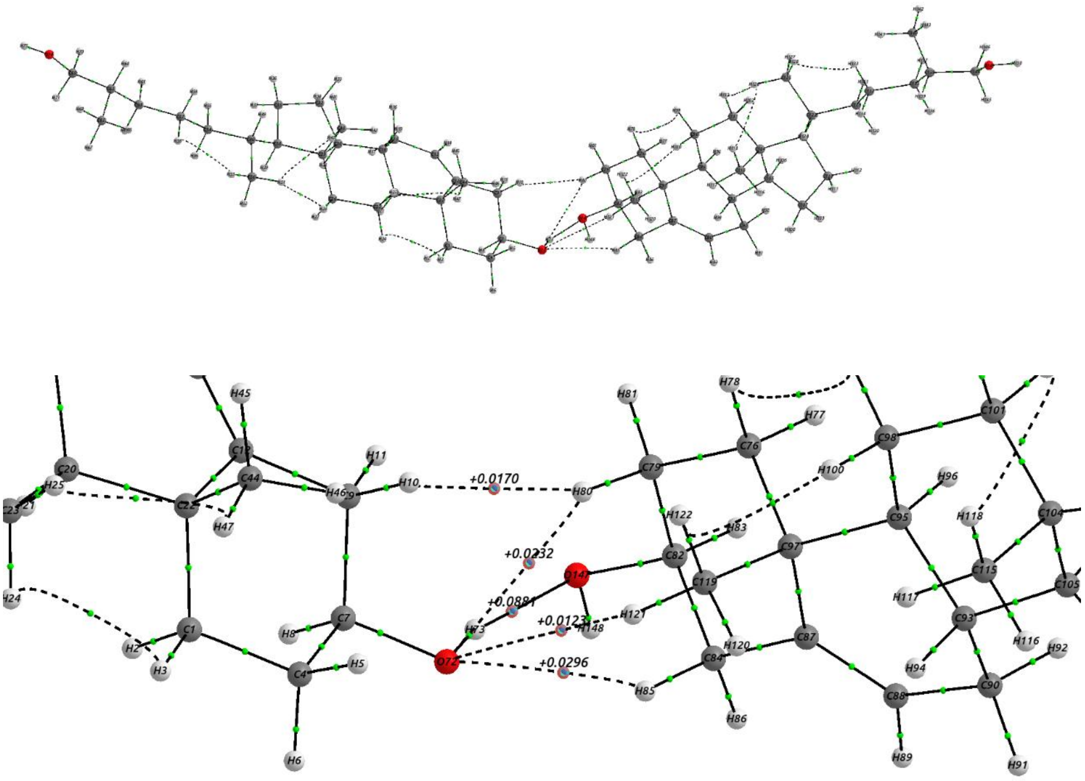

27-OH, 0--- 0---:

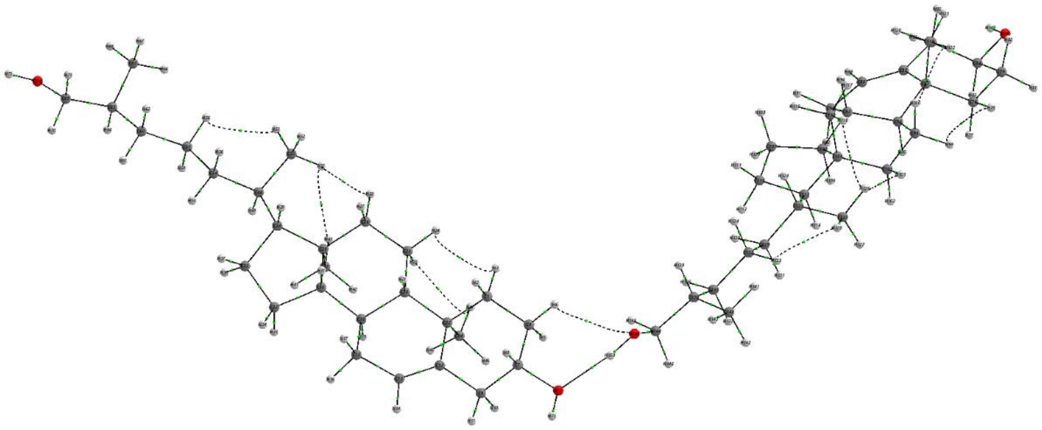

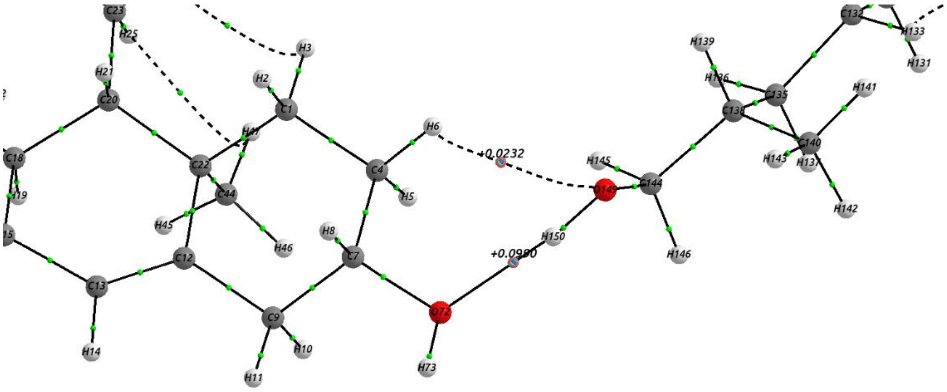

B) 25-OH, 0--- ---0:

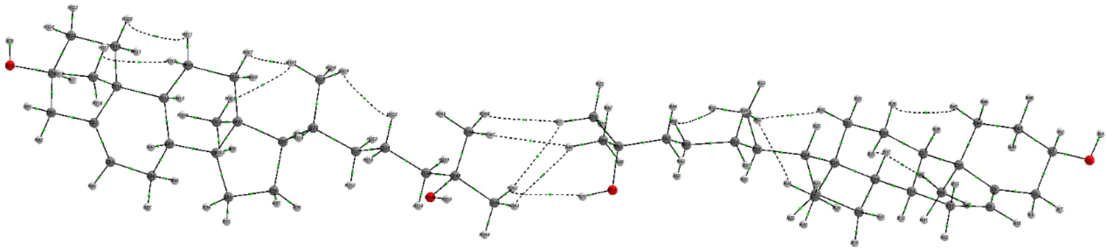

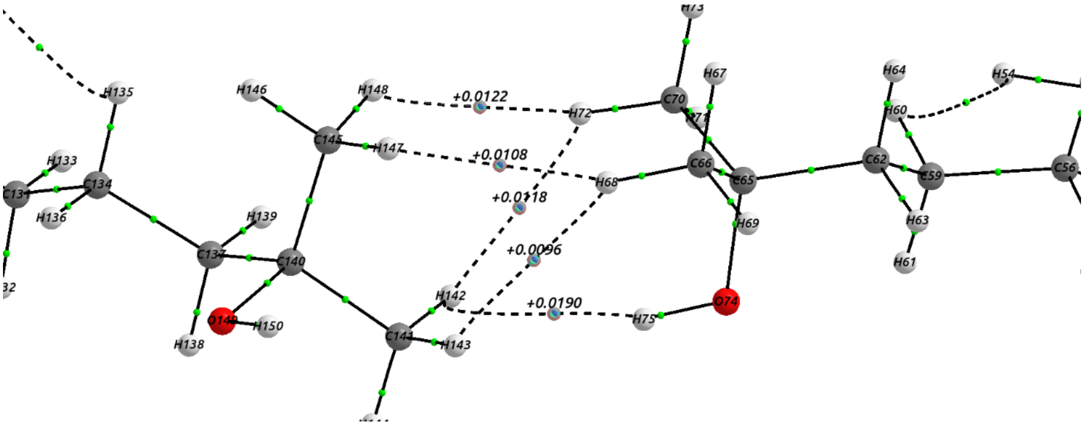

25-OH, ---0 0---:

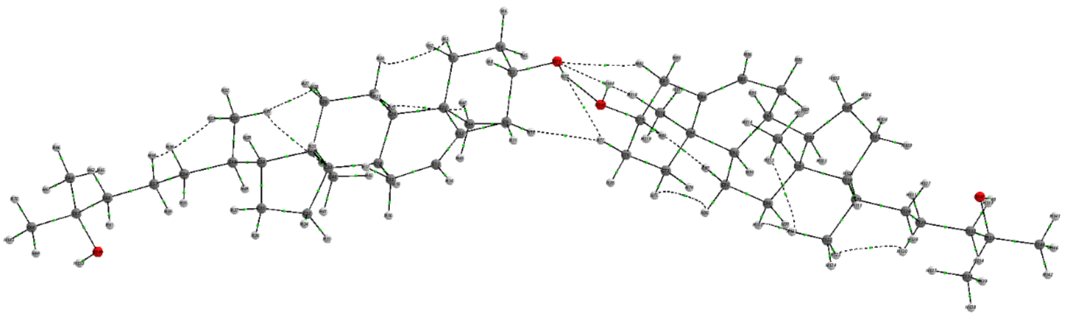

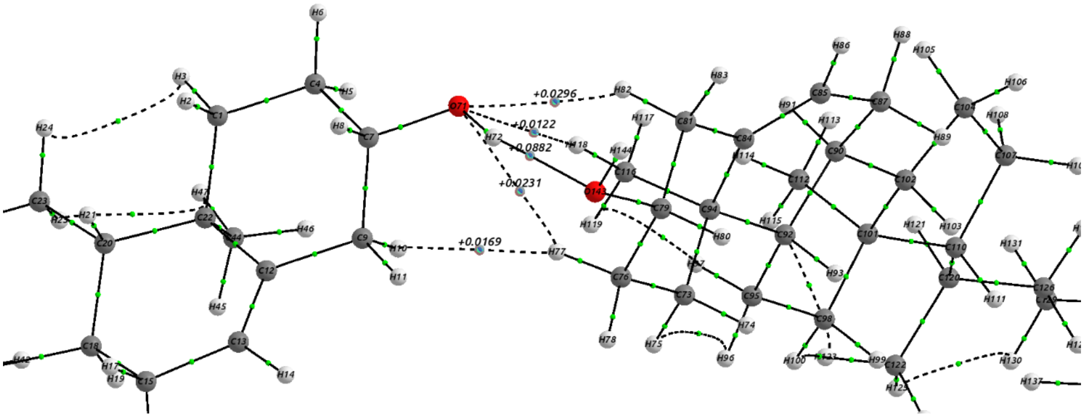

25-OH, 0--- 0---:

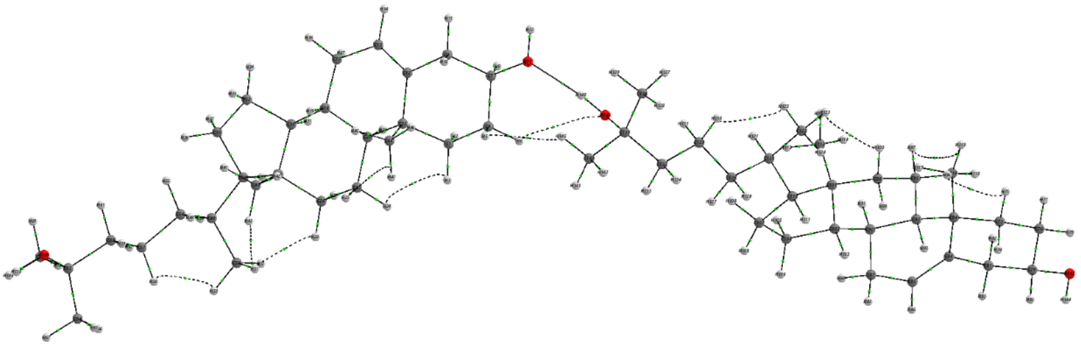

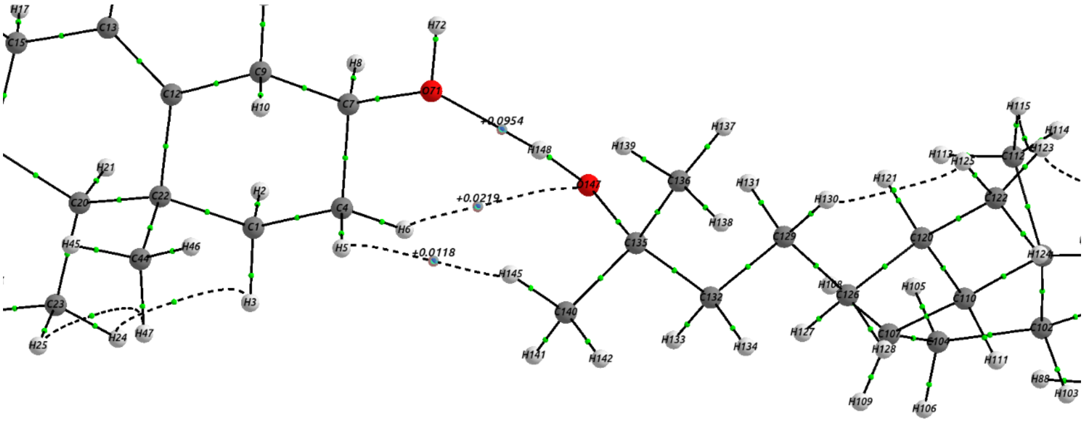

C) 24-OH, 0--- ---0:

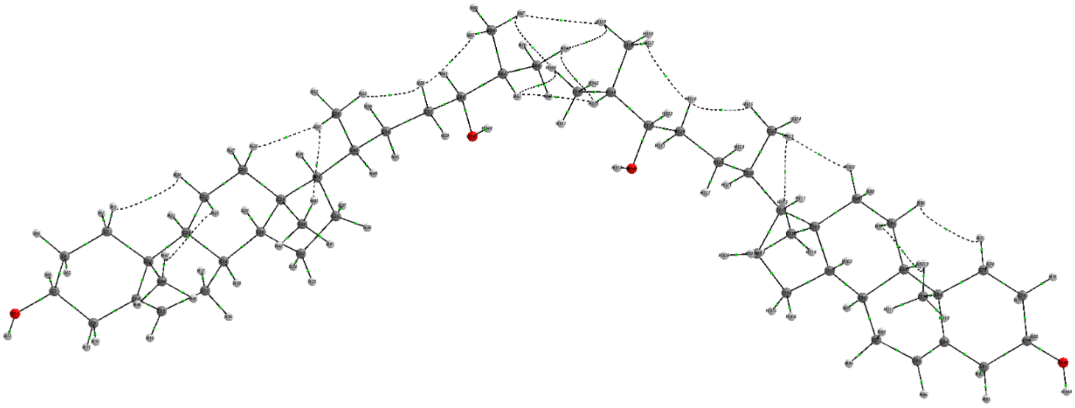

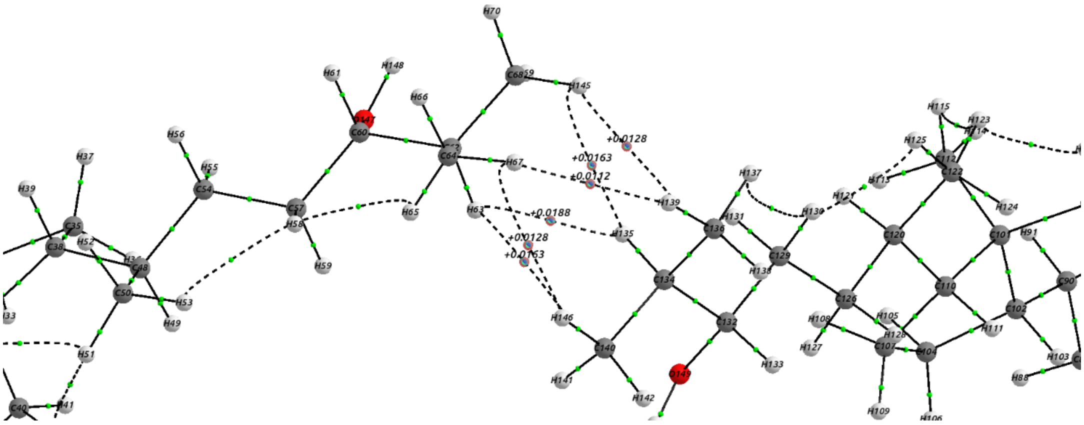

24-OH, ---0 0---:

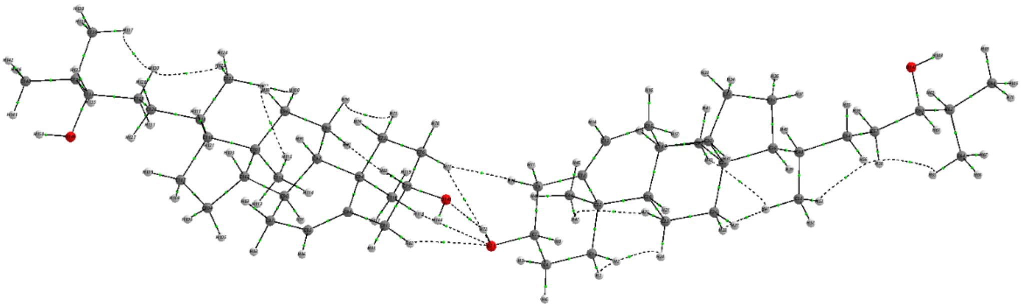

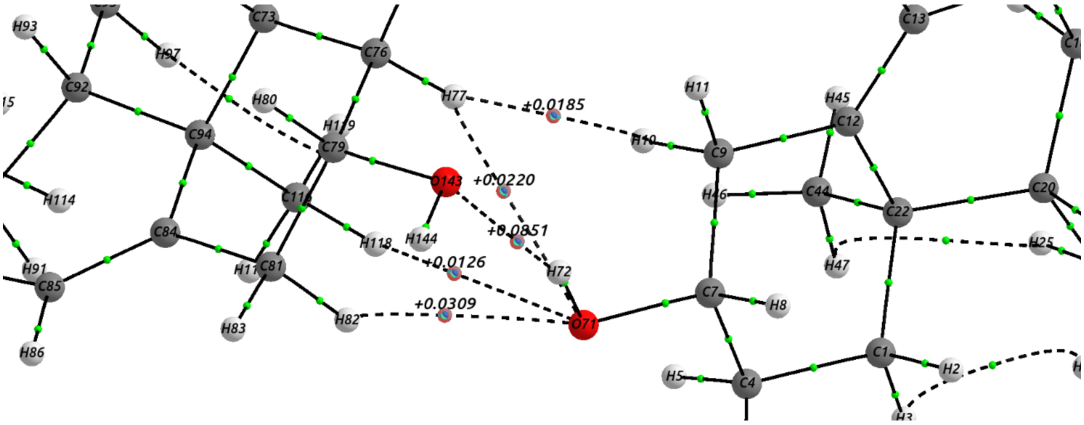

D) Chol, 0--- ---0:

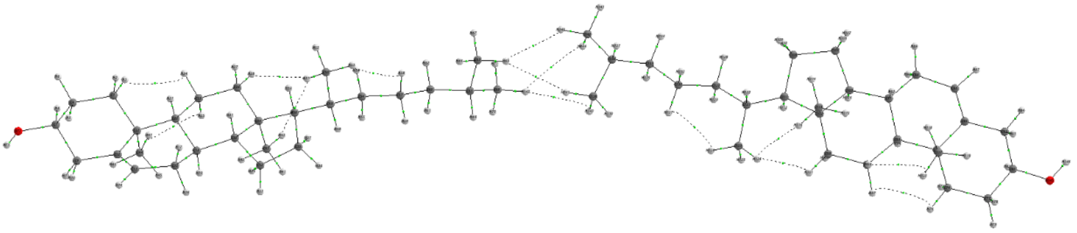

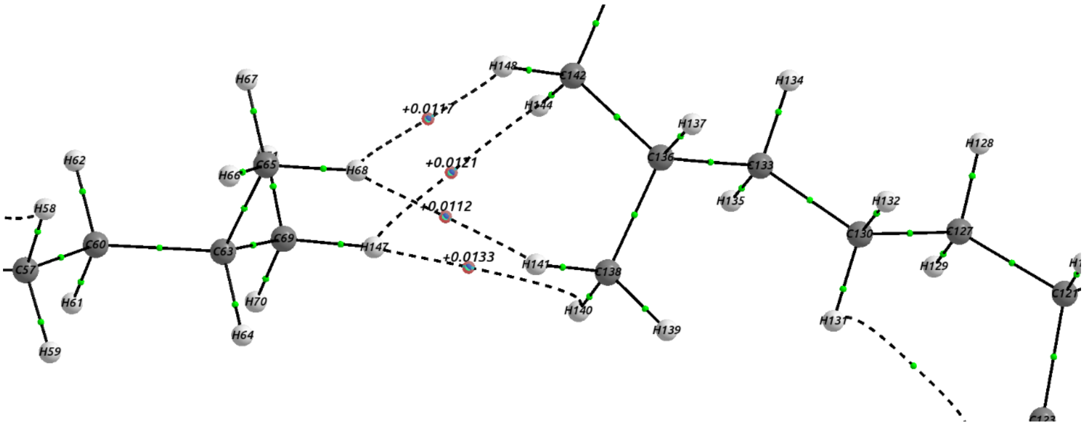

Chol, ---0 0---:

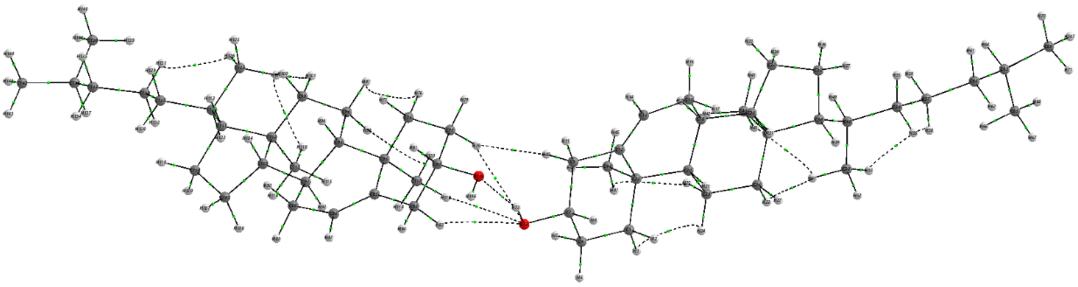

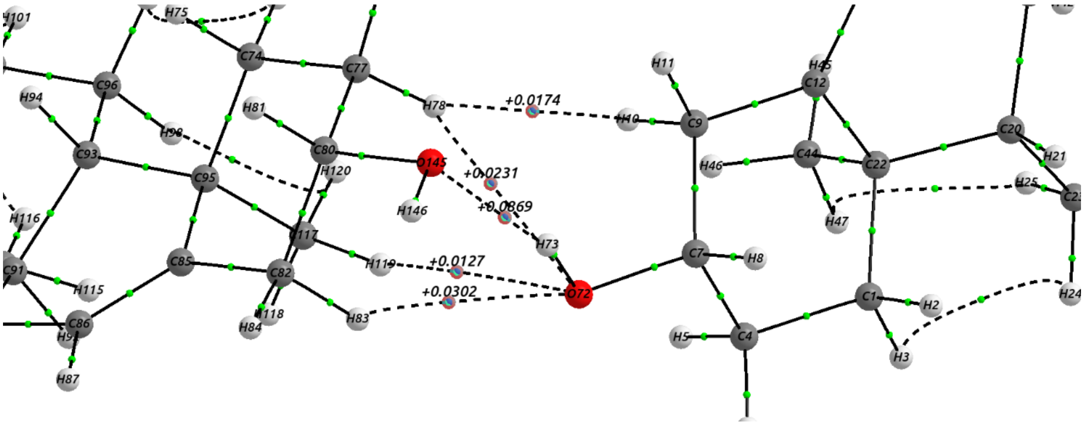

Figure S8. Bond paths from QTAIM analysis along with Laplacian values of the electron density in bond critical points for differently oriented dimers of 27-OH (A), 25-OH (B), 24-OH (C), and Chol (D).

A)

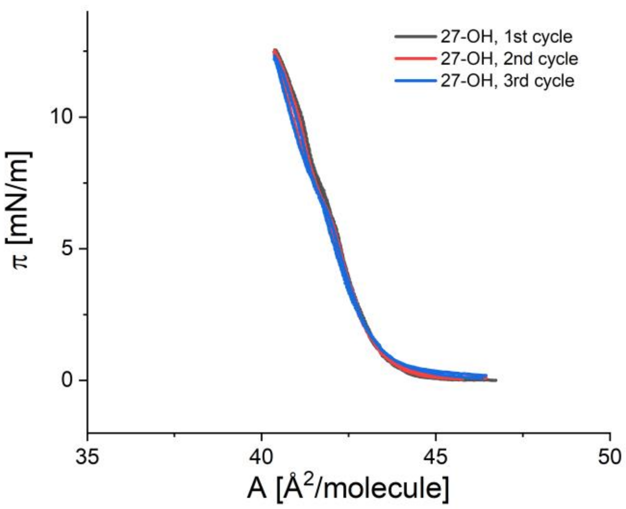

B)

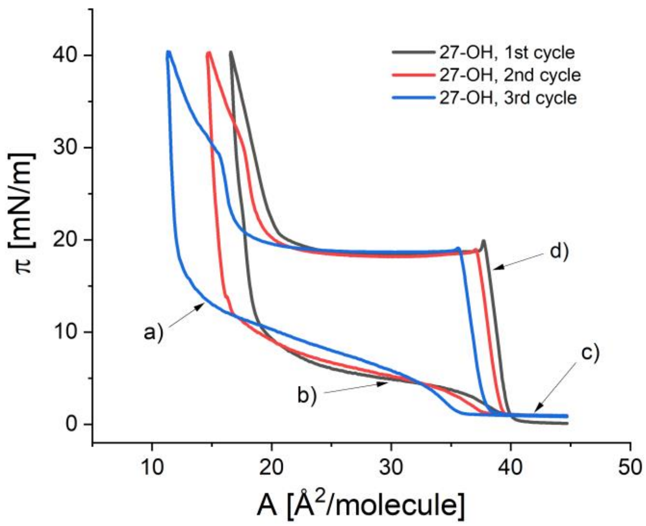

C)

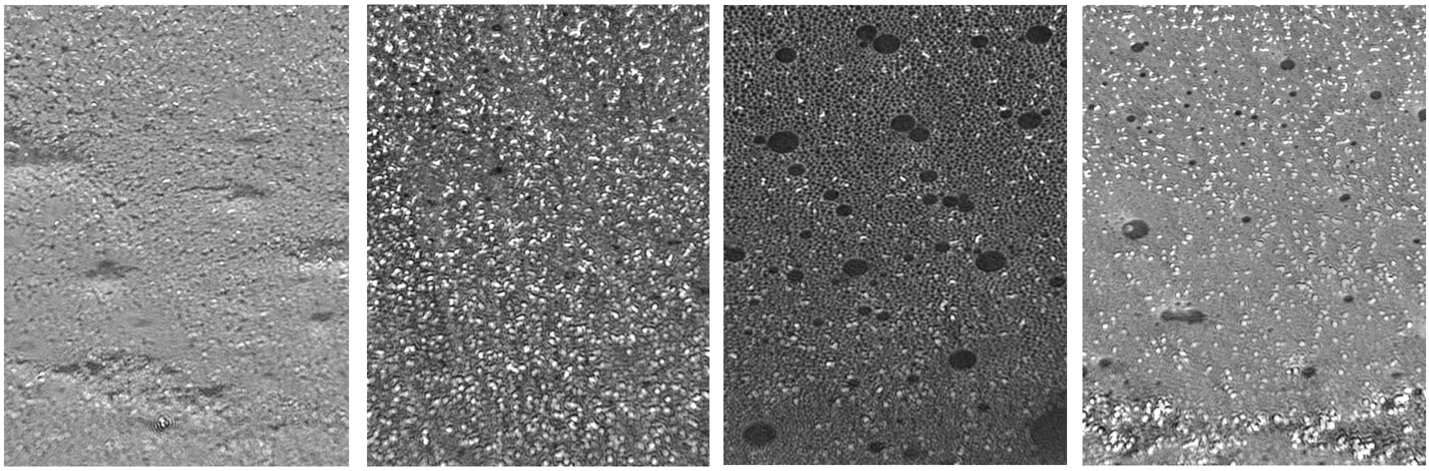

a) b) c) d)

Figure S9. Isotherms of continuous compression-expansion cycles registered for 27-OH at 20 °C for compression to surface pressure 15 mN/m (A) and mean molecular area 16 Å^2 (B), together with images of film textures (a-d) registered during experiment (C).

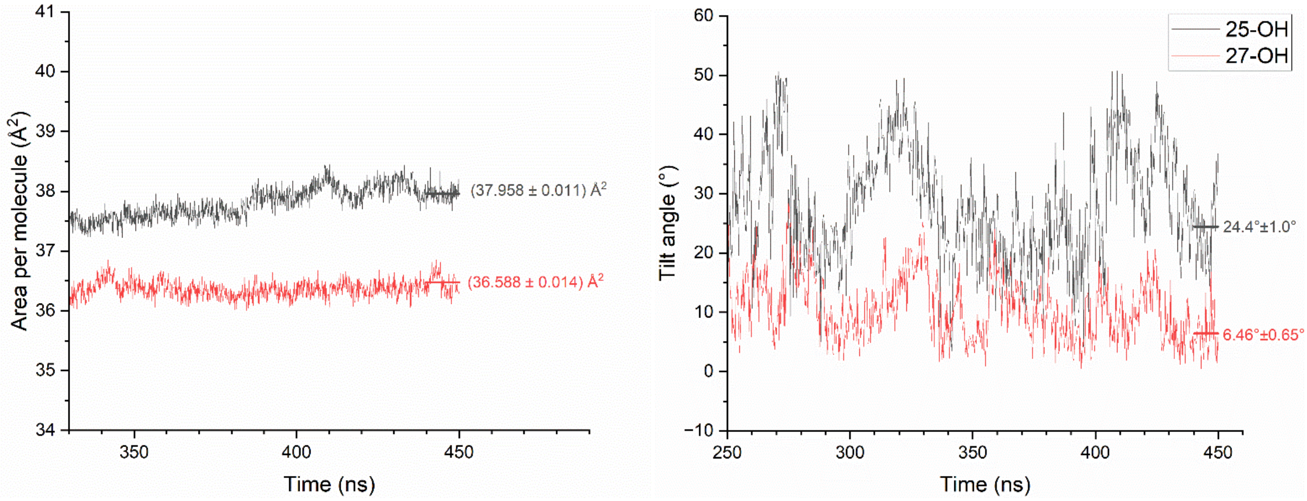

Figure S10. Averaged area per molecule (left) and tilt angle of oxysterol molecules (right) from the molecular dynamics of the 25-OH and 27-OH systems.

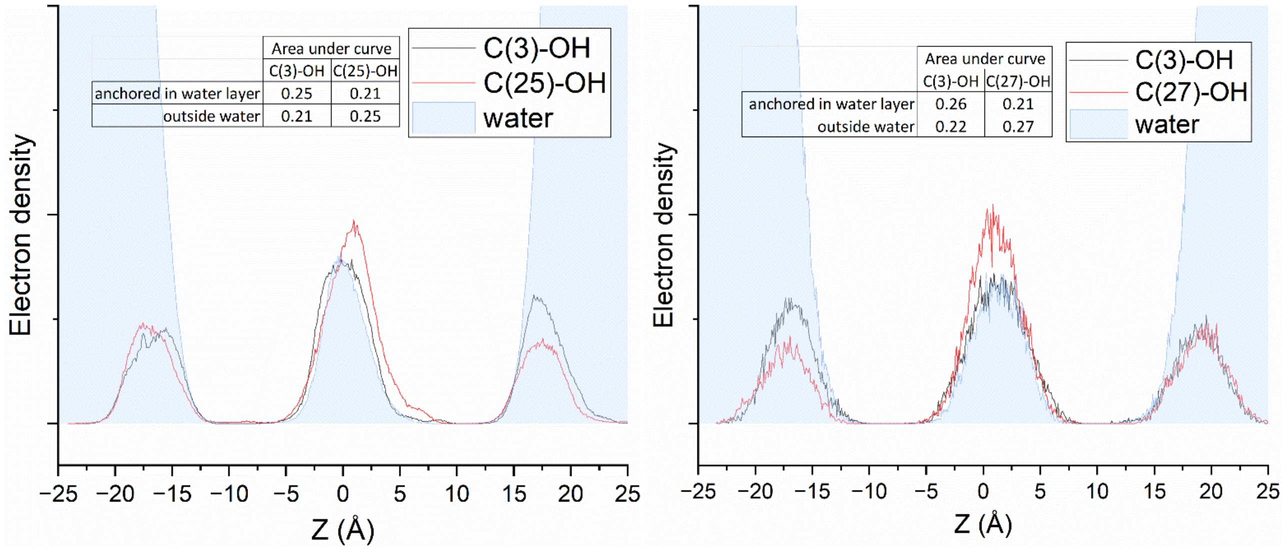

Figure S11. Electron density of the hydroxyl groups and water for the 25-OH and 27-OH systems along the Z coordinate.

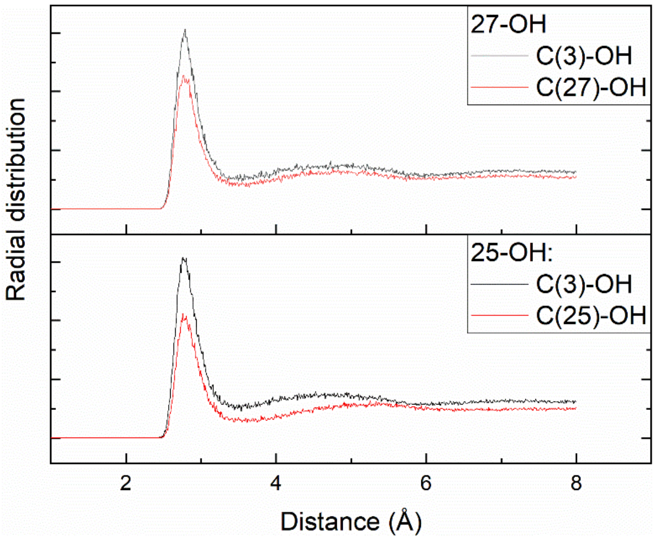

Figure S12. Radial distribution functions of the 25-OH and 27-OH systems, showing the density of water at a specified distance from the oxygen atom in the hydroxyl group.

A)

1. C(3)-OH…C(3)-OH
2. C(3)-OH…C(25)-OH
3. C(3)-OH…C(25)-OH
4. C(25)-OH…C(25)-OH
5. C(3)-OH…C(3)-OH
6. C(3)-OH…C(25)-OH
7. C(3)-OH…C(3)-OH
8. C(3)-OH…C(25)-OH

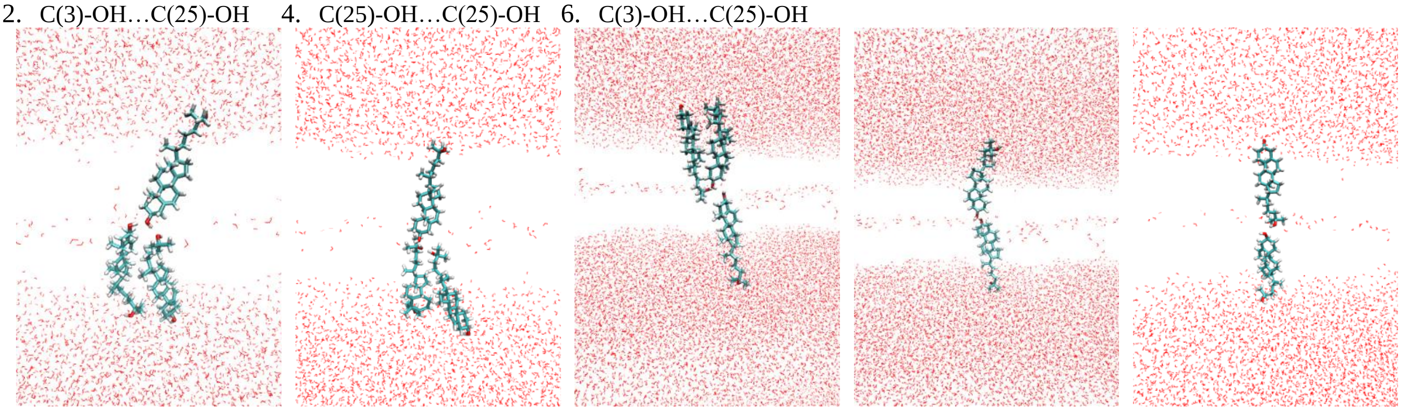

9. C(3)-OH…C(3)-OH
10. C(3)-OH…C(25)-OH
11. C(3)-OH…C(25)-OH
12. C(3)-OH…C(25)-OH

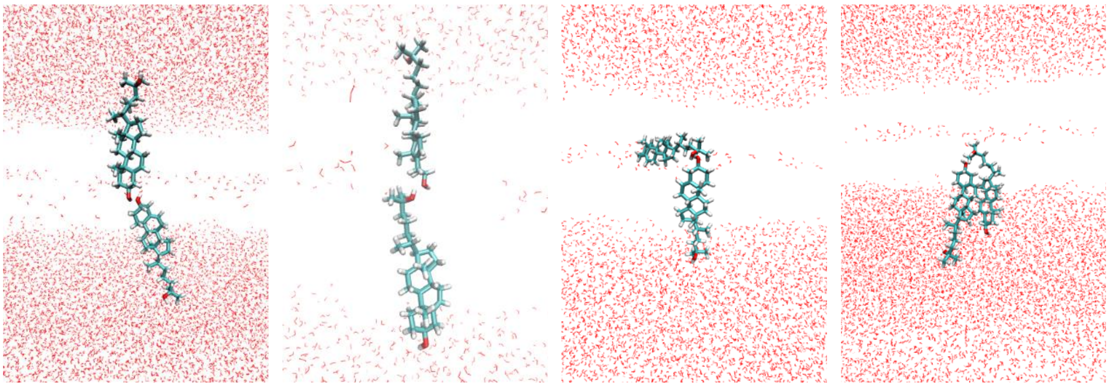

B)

1. C(3)-OH…C(3)-OH
2. C(3)-OH…C(3)-OH
3. C(3)-OH…C(3)-OH
4. C(3)-OH…C(27)-OH
5. C(3)-OH…C(27)-OH
6. C(3)-OH…C(3)-OH
7. C(3)-OH…C(3)-OH
8. C(3)-OH…C(3)-OH
9. C(3)-OH…C(27)-OH
10. C(3)-OH…C(3)-OH

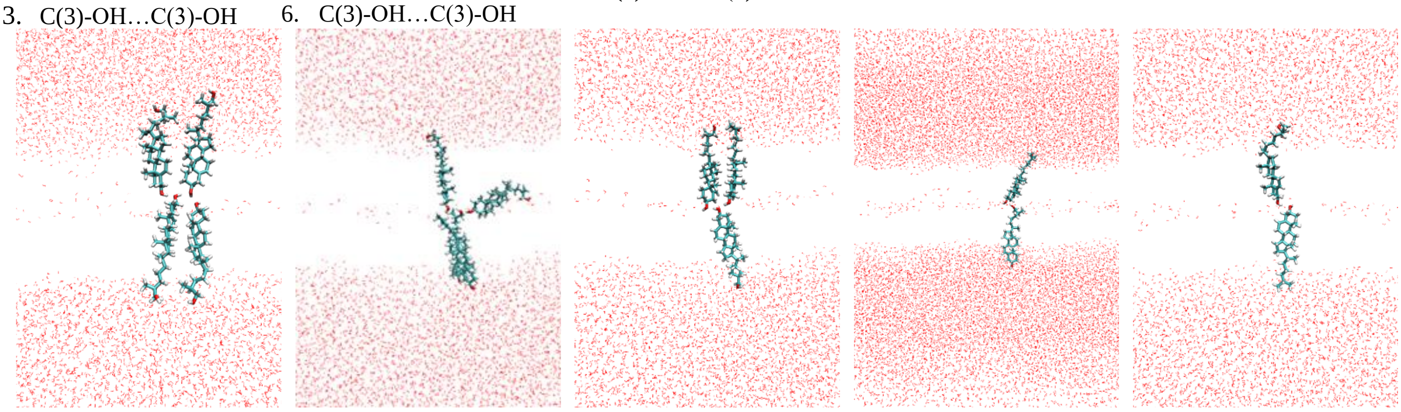

11. C(3)-OH…C(27)-OH

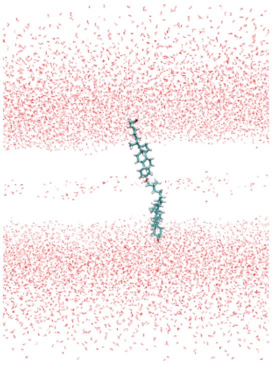

Figure S13. Snapshots of hydrogen bonded complexes of oxysterol molecules obtained from molecular dynamics simulations for the 25-OH (A) and 27-OH (B) systems.
